# Association of systemic immune biomarkers with metabolic dysfunction-associated steatotic liver disease: a cross-sectional study of NHANES 2007–2018

**DOI:** 10.3389/fnut.2024.1415484

**Published:** 2024-09-04

**Authors:** Yong Wang, Shude Chen, Chen Tian, Qi Wang, Zhihua Yang, Wieqi Che, Yike Li, Yang Luo

**Affiliations:** ^1^The First Clinical Medical School, Lanzhou University, Lanzhou, China; ^2^The Third Affiliated Hospital of Sun Yat-sen University, Guangzhou, China; ^3^Department of Health Policy and Management, School of Public Health, Lanzhou University, Lanzhou, China; ^4^Evidence-Based Social Science Research Center, School of Public Health, Lanzhou University, Lanzhou, China; ^5^Key Laboratory of Evidence Based Medicine and Knowledge Translation of Gansu Province, Lanzhou, China; ^6^Department of Epidemiology, School of Public Health, Cheeloo College of Medicine, Shandong University, Jinan, China; ^7^Department of Neurology, The First Hospital of Lanzhou University, Lanzhou, China; ^8^Key Laboratory of Biotherapy and Regenerative Medicine, Lanzhou, China

**Keywords:** systemic immune biomarkers, inflammation, metabolic dysfunction-associated steatotic liver disease, NHANES, cross-sectional study

## Abstract

**Objective:**

Numerous studies emphasize the pivotal role of inflammation in metabolic dysfunction-associated steatotic liver disease (MASLD) development. Some link specific systemic immune biomarkers (e.g., systemic immuno-inflammatory index [SII], neutrophil-to-albumin ratio [NPAR] and neutrophil-to-lymphocyte ratio [NLR]) to hepatic steatosis risk. However, the relevance of other markers like systemic immune-inflammation index [SIRI], platelet-to-lymphocyte ratio [PLR] and lymphocyte/monocyte ratio [LMR] in MASLD remains unclear. Limited literature covers all six markers together. This study aims to investigate the association between SII, SIRI, LMR, NLR, PLR, and NPAR and MASLD, assessing their predictive value.

**Methods:**

In this cross-sectional analysis of adults from NHANES (2007–2018), we investigated the relationship between six systemic immune biomarkers, stratified by quartiles: quartile1 (Q1), quartile2 (Q2), quartile3 (Q3) and quartile4 (Q4), and the outcome of MASLD assessed by Fatty Liver Index (FLI) and United States Fatty Liver Index (USFLI). Logistic regression and restricted cubic splines (RCS) were employed to assess the association between systemic immune biomarkers and MASLD risks. Propensity score matching controlled for potential confounders, and receiver operating characteristic (ROC) curve analysis evaluated the biomarkers’ predictive performances for MASLD. Subgroup and interaction analysis were conducted to explore the effects of systemic immune biomarkers on MASLD risks. Multicollinearity was quantified using the variance inflation factor.

**Results:**

In total, 14,413 participants were included and 6,518 had MASLD. Compared with non-MASLD, participants with MASLD had higher SII, SIRI, NLR, PLR, and NPAR (*p* < 0.001). SII, SIRI, NLR, and NPAR were further validated in the restricted cubic splines (RCS) regression model and identified as positive linear relationships (*p* for nonlinear >0.05). The prevalence of MASLD increased with the Q4 of SII [OR = 1.47, 95%CI (1.24, 1.74)], SIRI [OR = 1.30, 95%CI (1.09, 1.54)], NLR [OR = 1.25, 95%CI (1.04, 1.49)], PLR [OR = 1.29, 95%CI (1.09, 1.53)] and NPAR [OR = 1.29, 95%CI (1.09, 1.54)] compared to the Q1 after adjusting for the bias caused by potential confounders. However, the propensity score matching analysis only supported an association between the highest SII, SIRI, NLR NPAR and the risk of MASLD. The results of the subgroup analysis showed considerable robustness in the relationship.

**Conclusion:**

Higher SII, SIRI, NLR and NPAR were positively associated with a heightened risk of MASLD. NPAR showed the superior predictive value, followed by SII, SIRI and NLR. This needs to be validated in additional longitudinal studies and clinical trials.

## 1 Introduction

Metabolic dysfunction-associated steatotic liver disease (MASLD) constitutes a clinicopathologic syndrome characterized by diffuse steatosis (≥5%) (1), including simple fatty liver disease and its evolution steatohepatitis (NASH), liver fibrosis, cirrhosis and even liver cancer (2). The intricate pathogenesis of MASLD is intimately linked to factors such as insulin resistance, obesity, dyslipidemia, and genetic predisposition (3, 4). With the escalating incidence of obesity and diabetes, MASLD has emerged as a formidable public health challenge (5). Estes et al. (6) predicted that the global burden of MASLD will continue to increase based on a mathematical prediction model, which will be an important global health problem. The prevalence of MASLD in the United States has increased significantly over the past few decades, with approximately 30% of the population now affected by it (7). In view of the increasing risk and incidence of adverse outcomes of MASLD, early diagnosis, effective prevention and treatment of MASLD are necessary.

Many studies have shown that inflammation is a key factor in hepatic steatosis (8). Hepatic steatosis can manifest as a benign condition or progress to hepatocyte injury, triggering inflammation that activates immune cells. Infiltrating macrophages, T lymphocytes, neutrophils, and dendritic cells have the potential to induce liver inflammation and stimulate hepatic stellate cells, contributing to the progression of liver fibrosis (9). A cohort study conducted by Plessis demonstrated that heightened levels of TNF-α, IL8, and CCL3 were correlated with the severity of steatohepatitis. Moreover, the release of pro-inflammatory cytokines and chemokines by CD11c+ CD206+ macrophages and adipose tissue macrophages (ATM) significantly contributes to the pathogenesis of MASLD (10). A prior animal study suggested that the hepatocyte inflammasome could serve as a crucial link between non-alcoholic steatohepatitis (NASH) hepatocyte death and fibrotic stimulation. It may also function as a noninvasive indicator of inflammation (11). Participants with non-alcoholic steatohepatitis (NASH) exhibit elevated levels of inflammatory cytokines, potentially contributing to chronic inflammation and fostering disease progression. Furthermore, systemic inflammation is widely recognized as playing a significant role in the pathogenesis of advanced cirrhosis (12). The early detection and assessment of MASLD are imperative for effective management, monitoring of disease progression, and guiding treatment decisions. Although traditional liver biopsies are deemed the gold standard for chronic liver disease, their invasive, costly, and potentially hazardous nature underscores the need for noninvasive methods to identify the presence of MASLD. Such noninvasive approaches would offer significant benefits in terms of patient safety and cost-effectiveness.

The Systemic Immune-Inflammatory Index (SII) and the Systemic Inflammatory Response Index (SIRI) serve as crucial indicators of the systemic inflammatory response in organisms (13). The study revealed that in patients with malignant tumors, the Systemic Immune-Inflammatory Index (SII) objectively reflects a balance between inflammatory and immune responses. Meanwhile, the Systemic Inflammatory Response Index (SIRI) is regarded as a reliable indicator of the body’s chronic inflammatory state, capable of predicting the prognosis of patients with cancer and hypertension (14). In conclusion, SII and SIRI have been used as prognostic indicators in liver cancer research (15, 16). However, there is limited research on the influence of Systemic Immune-Inflammatory Index (SII) and Systemic Inflammatory Response Index (SIRI) on chronic non-alcoholic steatosis. Neutrophil-to-Lymphocyte Ratio (NLR), an easily measurable parameter, serves as a comprehensive reflection of two distinct yet complementary immune pathways, encompassing innate (neutrophilic) and adaptive (lymphocyte) cellular immune responses. NLR is associated with pro-inflammatory cytokines and can also function as an inflammatory marker (17). It has been studied as a factor related to disease severity and prognosis in many malignant and benign diseases (18). Platelet-to-Lymphocyte Ratio (PLR) is a novel hematologic inflammatory parameter that may provide insights into the development of inflammatory diseases to a certain extent. This method has been employed to predict the prognosis and incidence of malignant tumors, as well as cardiovascular and autoimmune diseases (19). Neutrophil-to-Albumin Ratio (NPAR) serves as a potent biomarker utilizing neutrophil counts and albumin values to offer an indicator of systemic inflammation. Previous studies indicate that NPAR can predict the occurrence of various conditions such as acute kidney injury, cardiogenic shock, myocardial infarction, and cancer (20). In conclusion, SII, SIRI, NLR, PLR, LMR and NPAR collectively serve as crucial indicators reflecting inflammation status. These indices hold potential value for predicting disease prognosis and morbidity.

Previous studies have identified a positive association between levels of SII (21), NPAR and NLR (22) and the risk of hepatic steatosis. However, the association of SIRI, PLR and LMR with patients suffering from MASLD remains unclear. Additionally, few studies have undertaken a comprehensive examination of all six crucial systemic immune biomarkers to delve into MASLD. This study aims to assess the correlation between these immune biomarkers and the presence of MASLD, comparing the effects of SII, SIRI, NLR, PLR, LMR, and NPAR on MASLD within the same population to determine their predictive value. Recognizing the pivotal roles of gender, age, physical activity (PA), hypertension, diabetes, and body mass index (BMI) in the initiation and development of MASLD, we further conducted a subgroup analysis to investigate the impact of systemic immune biomarkers on stratified MASLD. This research endeavors to offer novel insights to the scientific community, shedding light on the relationship between blood inflammatory markers and MASLD. Such insights aim to guide health management and the development of public health policies for populations at risk of relevant diseases.

## 2 Methods

A descriptive, cross-sectional, correlational study was designed for this investigation. The study’s reporting adhered to the Strengthening the Reporting of Observational Studies in Epidemiology (STROBE) checklist for cross-sectional studies (23).

### 2.1 Study sample

NHANES^1^ is a comprehensive program assessing the health and nutrition of the population. It utilizes a combination of interviews and physical examinations to gather demographic, dietary, physical examination, laboratory, and questionnaire data. The data for this study comes from the 2007–2008, 2009–2010, 2011–2012, 2013–2014, 2015–2016, and 2017–2018 NHANES survey cycles, all of which can be accessed on the official NHANES website. We chose these six data periods because they provide the most comprehensive details on FLI and USFLI. Additionally, these data periods are publicly available, whereas the latest dataset is restricted and inaccessible.

### 2.2 Definition of MASLD

Fatty Liver Index (FLI) and United States Fatty Liver Index (USFLI) ranged from 0 to 100 using the following formula (24, 25):


$$\begin{array}{l} \mathrm{FLI}=\left(e^{\begin{array}{l} 0.953*\ln\;\left(\mathrm{TG}\right)+0.139*\mathrm{BMI}+0.718*\ln\;\left(\mathrm{GGT}\right)\\ +0.053*\mathrm{waist circumference}-15.745 \end{array}}\right)/\\ \left(1+e^{\begin{array}{l} 0.953*\ln\;\left(\mathrm{TG}\right)+0.139*\mathrm{BMI}+0.718*\ln\;\left(\mathrm{GGT}\right)\\ +0.053*\mathrm{waist circumference}-15.745 \end{array}}\right)*100; \end{array}$$

$$\begin{array}{l} \mathrm{USFLI}=e^{\left(\begin{array}{l} 0.3458*\mathrm{Mexican American}-0.8073*\mathrm{non}-\mathrm{Hispanic black}\\ +0.0093*\mathrm{age}+0.6151*\mathrm{lnGGT}+0.0249*\mathrm{waist circumference}\\ +1.1792*\mathrm{insulin}+0.8242*\mathrm{lnGlucose}-14.7812 \end{array}\right)}/\\ \left(1+e^{\left(\begin{array}{l} 0.3458*\mathrm{Mexican American}-0.8073\\ {}*\mathrm{non}-\mathrm{Hispanicblack}+0.0093*\mathrm{age}+0.6151\\ {}*\mathrm{lnGGT}+0.0249*\mathrm{waist circumference}\\ +1.1792*\mathrm{insulin}+0.8242*\mathrm{lnGlucos}-14.7812 \end{array}\right)}\right)*100 \end{array}$$

(“non-Hispanic black” and “Mexican American” have a value of 1 if the participant is of that ethnicity and 0 if not of that ethnicity).

Hepatic steatosis was defined as a FLI ≥60 or a USFLI ≥30 (26). MASLD was defined as the presence of hepatic steatosis in the absence of (1) hepatitis B (positive hepatitis B surface antigen) or hepatitis C infection (positive hepatitis C antibody or HCV RNA); (2) the possibility of secondary liver disease caused by excessive alcohol consumption (more than 12 drinks in the past year, with all others considered non-drinkers; >1 alcoholic drink/day for women or >2 alcoholic drinks/day for men) and drug; (3) liver cancer; (4) autoimmune liver disease.

Advanced fibrosis was assessed by serological non-invasive fibrosis index, including the FIB-4 and NFS score, calculated using the following formula (27, 28):


FIB−4=(age∗AST)/(prattlet counts∗(SQRT(ALT)));

$$\begin{array}{l} \mathrm{NFS}=-1.675+0.037*\mathrm{age}+0.094*\mathrm{BMI}+1.13\\ {}*\mathrm{impaired fasting glucose}/\mathrm{diabetes}\;\left(\mathrm{yes}=1\mathrm{,no}=0\right)\\ +0.99*\mathrm{AST}/\mathrm{ALT}\;\mathrm{ratio}-0.013\\ {}*\mathrm{prattlet counts}-0.66*\mathrm{albumin}. \end{array}$$

A FIB-4 > 2.67 or NFS > 0.676 was defined as the presence of advanced fibrosis (29).

### 2.3 Exposure variable

Hematologic parameters were assessed following the NHANES CBC Profile using the Beckman Coulter Automated Hematology Analyzer DxH 900 (Beckman-Coulter, Brea, CA, United States). This analyzer performs red and white cell counts, and measures hemoglobin, hematocrit, and red blood cell indices. The Coulter VCS system is utilized for the white blood cell (WBC) differential. The Beckman Coulter Analyzer system employs an automatic dilution and mixing system for sample processing and a single-beam photometer for hemoglobinometry.

Lymphocyte, neutrophil, and platelet counts, expressed as ×10^3^ cells/μL, were measured using automated hematology analyzing devices. The following formulas were used to calculate immune-inflammatory markers: (1) Systemic Immune-Inflammation Index (SII) = platelet count * neutrophils count/lymphocytes count; (2) Systemic Inflammation Response Index (SIRI) = neutrophils count * monocyte count/lymphocytes count; (3) Lymphocyte-to-Monocyte Ratio (LMR) = lymphocytes count/monocyte count; (4) Neutrophil-to-Lymphocyte Ratio (NLR) = neutrophils count/lymphocytes count; (5) Platelet-to-Lymphocyte Ratio (PLR) = platelet count/lymphocytes count; (6) Neutrophil-to-Albumin Ratio (NPAR) = neutrophils count (%)/albumin (g/dL).

### 2.4 Study covariates

We aimed to reduce potential confounding bias in our analysis by selecting covariates based on previous research and clinical plausibility. The selected covariates can be categorized as follows:

1. Demographic information: Gender (male or female); Age; Race/ethnicity (Mexican American, other Hispanic, non-Hispanic White, non-Hispanic Black, other including multi-racial); Education level (below high school, high school above); Poverty level expressed as poverty–income ratio (PIR) (Bounded by 1.3, subjects with PIR < 1.3 are defined as poor) (30); Health insurance; Marital status.

2. Clinical measurements: Body Mass Index (BMI) (BMI < 25 kg/m^2^ is considered normal/underweight, 25 ≤ BMI < 30 kg/m^2^ is considered overweight, BMI ≥ 30 kg/m^2^ is considered obesity); Hypertension (defined as self-reported high blood pressure, use of antihypertensive medications, or mean systolic blood pressure ≥140 mmHg and/or mean diastolic blood pressure ≥90 mmHg); Waist circumference (WC).

3. Biomedical test results: Diabetes was based on the fulfillment of the American Diabetes Association criteria (31) for diabetes diagnosis (fasting plasma glucose concentration ≥126 mg/dL, 2 h plasma glucose ≥200 mg/dL during an oral glucose tolerance test, or Hemoglobin A1c (HbA1c) ≥6.5%) or an answer of “yes” to any of the following questions: (1) Other than during pregnancy, have you ever been told by a doctor or other health professional that you have diabetes or sugar diabetes? (2) Are you taking insulin now? (3) Are you taking diabetes pills to lower your blood glucose?

4. Self-reported lifestyle information: Smoking status [defined as smoking more than 100 cigarettes in entire life, with all others considered non-smokers (32)]; Alcohol consumption [defined as having more than 12 drinks in the past year, with all others considered non-drinkers (33)]; Cardiovascular disease (defined as self-report of coronary heart disease, angina, myocardial infarction, stroke, or congestive heart failure). Physical activity (PA) patterns were also assessed. NHANES defined exercises that cause large increases in breathing or heart rate as vigorous-intensity activity, while moderate-intensity activity was defined as exercises that cause relatively small increases in breathing or heart rate (34). Moderate-to-vigorous physical activity (MVPA) minutes per week were calculated using the formula: MVPA minutes per week = [moderate-intensity activity minutes × moderate-intensity days] + [vigorous-intensity activity minutes × vigorous-intensity days] (35). Based on the 2018 Physical Activity Guidelines for Americans (36), PA patterns were classified into two groups: insufficiently active group (MVPA < 150 min/wk) and sufficiently active group (MVPA ≥ 150 min/wk), respectively (37).

### 2.5 Statistical analysis

Descriptive statistics were employed to compare the clinical and demographic characteristics of participants. Continuous variables were presented as mean ± standard deviation, while categorical variables were expressed as frequency (percentage). The normality of continuous variables was assessed using the Kolmogorov–Smirnov normality test. Normally distributed variables were described with mean ± standard deviation (SD), and non-normally distributed variables were presented as median (interquartile range). Student’s *t*-test was applied to compare mean levels between MASLD and non-MASLD group for normally distributed variables. The Mann–Whitney U test was employed for non-normally distributed variables. Chi-square tests were used to assess differences in continuous and categorical variables.

Restricted cubic spline (RCS) was used to explore dose–response relationship between systemic immune biomarkers and MASLD (38). Based on Harrell’s recommendation (39), we selected four knots (5th, 35th, 65th, and 95th) to smooth the curve (40). A logistic regression model was employed to assess the association between six systemic immune biomarkers and MASLD. Three models were analyzed to enhance the robustness of the results. Model 1 was the unadjusted model. Model 2 was adjusted for gender, age, race/ethnicity, PIR, education, marital status, and health insurance. Model 3 included further adjustments for tobacco use, alcohol use, hypertension, diabetes, cardiovascular disease, waist circumference (WC), physical activity (PA), body mass index (BMI), triglycerides (TG), high-density lipoprotein (HDL), alanine aminotransferase (ALT), aspartate aminotransferase (AST), and gamma-glutamyl transferase (GGT). The variance inflation factor (VIF) was used to test for multicollinearity between variables, the values for each variable is less than or equal to 5 was taken as no similarity (41). *p* values for trends were calculated using the tertiles median value as a quasi-continuous variable in the model. Missing values (PIR, education, marital status, health insurance, tobacco use, alcohol use and BMI) were imputed using the Multiple Imputation by Chained Equations (MICE). MICE is a general approach for imputing multivariate data, which replaces missing values with plausible values drawn from a distribution specifically modeled for each missing entry (42). The function generates an *m* number of imputed datasets [*m* = 5 in this study (43)] which differ in the imputed values. Then, a binary logistic regression was conducted using the data of all the imputed datasets. We conducted different models including all adjusted confounding variables for Model 3, as independent predictors, and we chose the model that best adjusted to the data (44).

Additionally, subgroup analysis were performed based on sex (men and women), age groups (<45 and ≥45), hypertension (no and yes), diabetes (no and yes), high cholesterol (no and yes), BMI (<30 kg/m^2^ and ≥30 kg/m^2^), and PA (insufficiently and sufficiently) to test the robustness and explore potential variations. An interaction test was used to assess the heterogeneity of the relationship between different subgroups (45). To reduce the probability of committing a type I error due to the high number of subgroup comparisons, Bonferroni correction was used (46). Because multiple comparisons in the 6 subgroups were performed 6 times respectively, the *p* value lesser than 0.05/6 (0.0083) was accepted for statistical significance after Bonferroni correction (47).

To assess the systemic immune biomarkers as predictors of MASLD, receiver operating characteristic (ROC) curves were calculated to evaluate their ability to distinguish between participants with and without MASLD. ROC curves use continuous variables to predict binary outcomes and serve as a useful tool for testing the performance of clinical trials in correctly differentiating outcomes (48). The area under the ROC curve (AUROC) quantifies the accuracy of the test, ranging from 0.5 (discrimination no better than chance) to 1.0 (perfect discrimination). AUROC values are interpreted as follows: non-informative/equal to chance (AUC = 0.5), less accurate (0.5 < AUC ≤ 0.7), moderately accurate (0.7 < AUC ≤ 0.9), highly accurate (0.9 < AUC < 1.0), and a perfect discriminatory test (AUC = 1.0) (49). The calibration of the model was also estimated using the Hosmer–Lemeshow goodness-of-fit test. A test statistic greater than 0.05 in the Hosmer–Lemeshow goodness-of-fit test indicates that the model is a good fit (50). The optimal cutoff value of the inflammation-related index level was determined based on the Youden index using the receiver operating characteristics curve (ROC).

Additionally, we conducted a sensitivity analysis, employing multiple imputations of chained equations to address missing values for certain variables. Propensity Score Matching (PSM) methods were utilized to adjust for baseline confounding variables between the MASLD and non-MASLD groups, ensuring more accurate conclusions. In this regard, a multivariate logistic regression analysis was employed to determine each participant’s propensity score based on the aforementioned study covariates. The MASLD and non-MASLD groups were then matched in a 1:1 ratio using nearest neighbor matching with a caliper width set at 0.2 logits standard deviation for the propensity score. This specific value was chosen as it minimizes the mean square error in estimating treatment effects across various cases. Propensity scoring achieved a balanced diagnosis, ensuring no significant differences in covariates between the two groups (normalized difference of all covariates <0.1).

All statistical analysis were performed using IBM SPSS software Version 27 (51) and R software Version 4.2.1 (52). Odds ratio (OR) with 95% Confidence Interval (CI) were calculated using logistic regression analysis. Statistical significance was defined as *p* < 0.05.

## 3 Results

**Figure 1:**
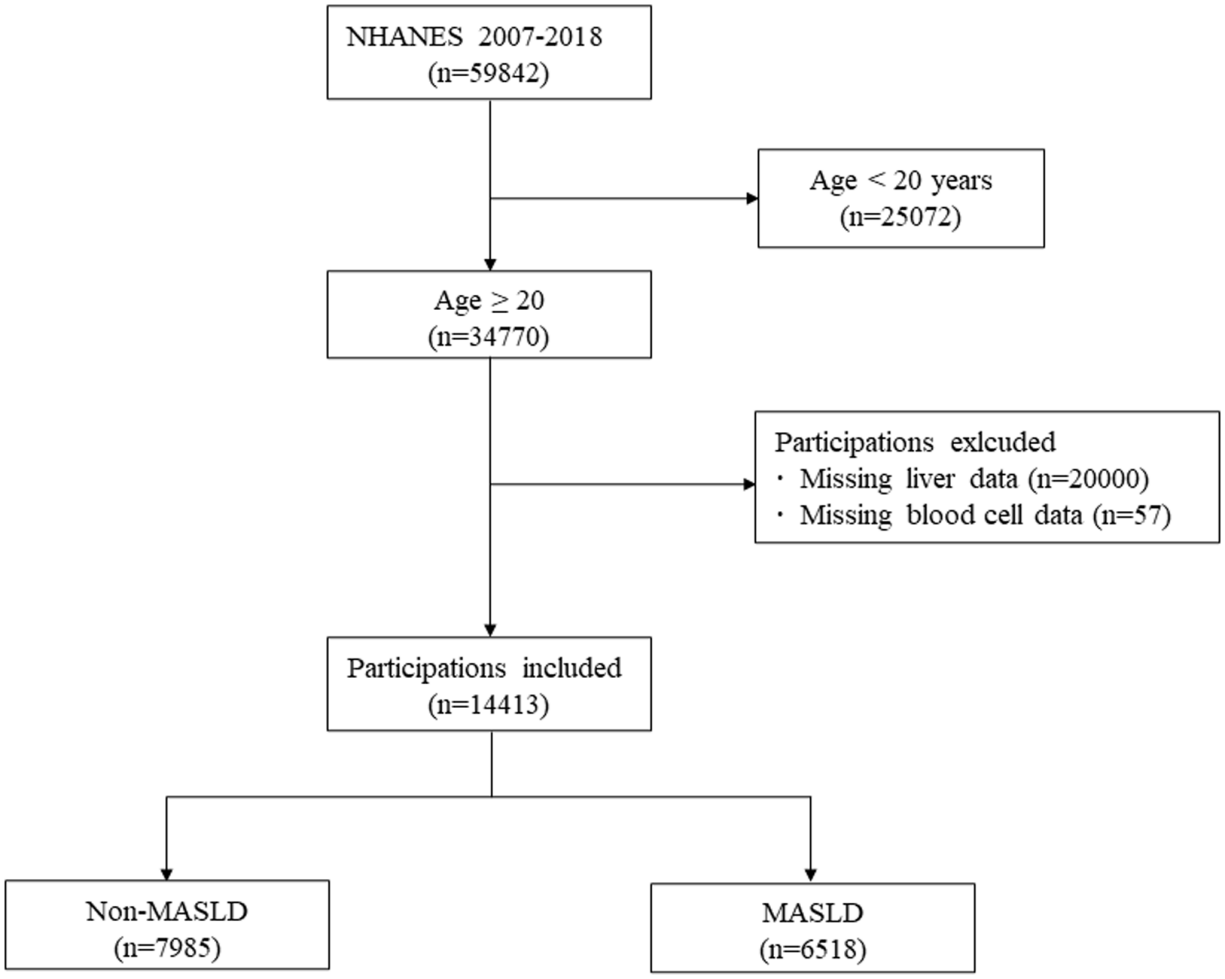
Flow chart of selecting eligible participants from NHANES 2007–2018.

Among a total of 59,842 subjects in the NHANES 2007–2018, we included 34,770 subjects aged ≥20 years (53). Of these, 45,129 subjects who met the following criteria were excluded: (1) Age < 20 (*n* = 2,572); (2) Missing data of liver (*n* = 20,000); (3) Missing data of complete blood cell (*n* = 57). Finally, 14,413 subjects were included in the analysis, of which 6,518 were participants with MASLD, and 7,985 were participants without MASLD (Figure 1).

### 3.1 Characteristics of participants

Table 1 summarizes the baseline characteristics of 14,413 participants in this analysis. 54.78% were male, and 45.22% female, average age 49.76 years. 6,518 had non-MASLD, 7,895 had MASLD. Participants of advanced age, with obesity, higher education levels, sociability, and lower income, exhibited a heightened risk of MASLD (all *p* < 0.05). In terms of physical health, participants with insufficient physical activity, hypertension, diabetes, and cardiovascular history exhibited a heightened risk of MASLD (all *p* < 0.05). Compared to non-MASLD, Participants with MASLD had higher SII (492.59 [311.21] vs. 539.77 [474.60]; *p* < 0.05), SIRI (1.10 [0.85] vs. 1.25 [0.96]; *p* < 0.05), NLR (2.08 [1.15] vs. 2.20 [1.23]; *p* < 0.05), and NPAR (1.34 [0.27] vs. 1.41 [0.27]; *p* < 0.05).

**Table 1:** Demographic and general health characteristics of participants with and without MASLD from the 2007–2018 National Health and Nutrition Examination Survey.

Characteristic	Total	Non-MASLD	MASLD	*p*-value
	(*n* = 14,413)	(*n* = 7,895)	(*n* = 6,518)	
Age (years, mean ± SD)	49.76 ± 17.51	48.16 ± 18.32	51.70 ± 16.28	<0.001^*^^b^
Gender, *n* (%)				
Male	7,008 (48.62)	3,547 (44.93)	3,461 (53.10)	<0.001^*^^a^
Female	7,405 (51.38)	4,348 (55.07)	3,057 (46.90)	
Race/Ethnicity, *n* (%)				
Mexican American	2,225 (15.44)	965 (12.22)	1,260 (19.33)	<0.001^*^^a^
Other Hispanic	1,595 (11.07)	835 (10.58)	760 (11.66)	
Non-Hispanic White	5,948 (41.27)	3,239 (41.03)	2,709 (41.56)	
Non-Hispanic Black	2,868 (19.90)	1,586 (20.09)	1,282 (19.67)	
Other Race	1,777 (12.33)	1,270 (16.09)	507 (7.78)	
PIR, *n* (%)				
Poor (<1.3)	9,711 (67.38)	5,455 (69.09)	4,256 (65.30)	<0.001^*^^a^
Inpoor (≥1.3)	3,702 (25.69)	1,440 (18.24)	2,262 (34.70)	
Education, *n* (%)				
<High school	3,593 (24.93)	1,772 (22.44)	1,821 (27.94)	<0.001^*^^a^
≥High school	10,820 (75.07)	6,123 (77.56)	4,697 (72.06)	
Marital status, *n* (%)				
Never married	2,567 (17.81)	1,592 (20.16)	975 (14.96)	<0.001^*^^a^
Widowed/Divorced/Separated	3,151 (21.86)	1,619 (20.51)	1,532 (23.50)	
Married/Living with partner	8,695 (60.33)	4,684 (59.33)	4,011 (61.54)	
Health insurance, *n* (%)				
Yes	11,273 (78.21)	6,170 (78.15)	5,103 (78.29)	0.839^a^
No	3,140 (21.79)	1,725 (21.85)	1,415 (21.71)	
BMI, *n* (%)				
<24.9 kg/m^2^	4,218 (29.27)	4,036 (51.12)	182 (2.79)	<0.001^*^^a^
25–29.9 kg/m^2^	4,794 (33.26)	3,208 (40.63)	1,586 (24.33)	
≥30 kg/m^2^	5,401 (37.47)	651 (8.25)	4,750 (72.88)	
Tobacco use, *n* (%)				
Yes	6,399 (44.40)	3,265 (41.36)	3,134 (48.08)	<0.001^*^^a^
No	8,014 (55.60)	4,630 (58.64)	3,384 (51.92)	
Alcohol use, *n* (%)				
Yes	7,676 (53.26)	4,347 (55.06)	3,329 (51.07)	<0.001^*^^a^
No	6,737 (46.74)	3,548 (44.94)	3,189 (48.93)	
WC				
<102 for male or <88 for female	6,209 (43.08)	5,354 (67.82)	855 (13.12)	<0.001^*^^a^
≥102 for male or ≥88 for female	8,204 (56.92)	2,541 (32.18)	5,663 (86.88)	
Cardiovascular diseases, *n* (%)				
Yes	1,0702 (74.25)	6,170 (78.15)	4,532 (69.53)	<0.001^*^^a^
No	3,711 (25.75)	1,725 (21.85)	1986 (30.47)	
Hypertension, *n* (%)				
Yes	8,406 (58.32)	5,382 (68.17)	3,024 (46.39)	<0.001^*^^a^
No	6,007 (41.68)	2,513 (31.83)	3,494 (53.61)	
Diabetes, *n* (%)				
Yes	1,1,607 (80.53)	7,102 (89.96)	4,505 (69.12)	<0.001^*^^a^
No	2,806 (19.47)	793 (10.04)	2,013 (30.88)	
Physical activity, *n* (%)				
Insufficiently	5,681 (39.42)	2,811 (35.6)	2,870 (44.03)	<0.001^*^^a^
Sufficiently	8,732 (60.58)	5,084 (64.4)	3,648 (55.97)	
TG				
Yes	3,018 (20.94)	702 (8.89)	2,316 (35.53)	<0.001^*^^a^
No	11,395 (79.06)	7,193 (91.11)	4,202 (64.47)	
HDL				
Yes	424 (2.94)	368 (4.66)	56 (0.86)	<0.001^*^^a^
No	13,989 (97.06)	7,527 (95.33)	6,462 (99.14)	
ALT, (mean ± SD)	25.01 ± 19.47	20.98 ± 12.04	29.89 ± 24.89	<0.001^*^^b^
AST, (mean ± SD)	25.35 ± 20.34	23.71 ± 14.07	27.33 ± 25.85	<0.001^*^^b^
GGT, (mean ± SD)	29.67 ± 39.44	20.985 ± 21.75	40.36 ± 51.56	<0.001^*^^b^
SII, (mean ± SD)	513.93 ± 394.28	492.59 ± 311.21	539.77 ± 474.60	<0.001^*^^b^
SIRI, (mean ± SD)	1.17 ± 0.91	1.10 ± 0.85	1.25 ± 0.96	<0.001^*^^b^
LMR, (mean ± SD)	4.16 ± 1.84	4.14 ± 1.78	4.17 ± 1.91	0.412^b^
NLR, (mean ± SD)	2.13 ± 1.18	2.08 ± 1.15	2.20 ± 1.23	<0.001^*^^b^
PLR, (mean ± SD)	0.01 ± 0.01	0.01 ± 0.01	0.01 ± 0.01	0.029^*^^b^
NPAR, (mean ± SD)	1.37 ± 0.27	1.34 ± 0.27	1.41 ± 0.27	<0.001^*^^b^

ALT, alanine aminotransferase; AST, aspartate aminotransferase; BMI, body mass index; GGT, Gamma-Glutamyl transferase; HDL, high density; PIR, poverty income ratio; LMR, Lymphocyte-to-Monocyte ratio; NLR, Neutrophil-to Lymphocyte ratio; NPAR, Neutrophil-to-Albumin ratio; PIR, poverty income ratio; PLR, Platelet-to-Lymphocyte ratio; SII, systemic immune-inflammation index; SIRI, systemic inflammation response index; TG, triglyceride; WC, waist circumference. *p < 0.05 is considered statistically significant.

a Chi-square test was used to compare the percentage between participants with and without MASLD.

b Mann–Whitney U test was used to compare the median values between participants with and without MASLD.

### 3.2 Linear relationship between systemic immune biomarkers and MASLD

**Figure 2:**
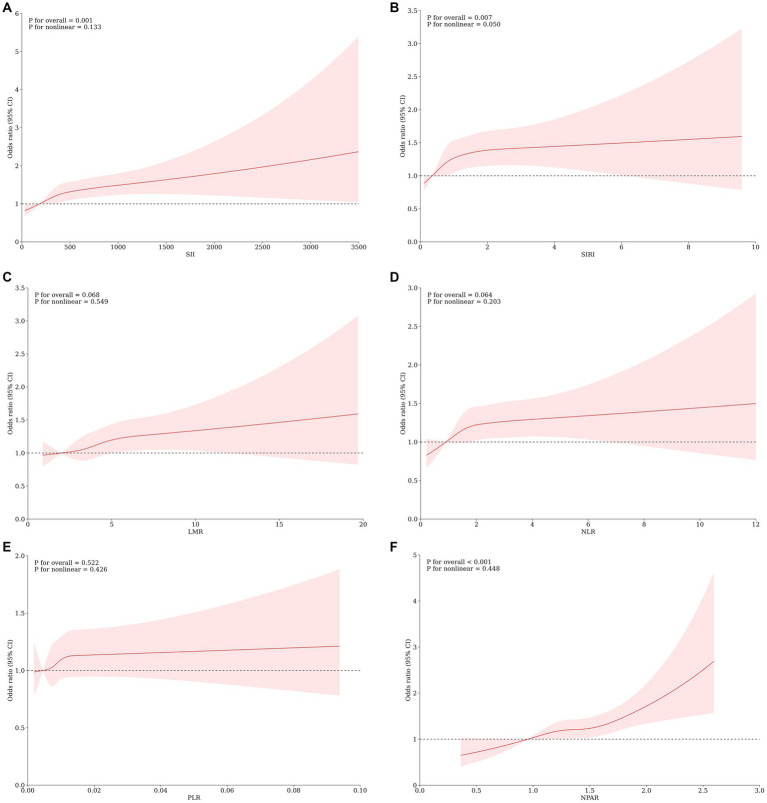
Association Between SII **(A)** SIRI **(B)**, LMR **(C)**, NLR **(D)**, PLR **(E)**, NPAR **(F)** and the risk of MASLD using a restricted cubic spline regression model. OR, odds ratio; CI, confidence interval. *p < 0.05 is considered statistically significant. The model was conducted with 4 knots at the 5th, 35th, 65th, and 95th percentiles of systemic immune biomarkers (reference is the 1st quartile). Data were fitted by a logistic regression model. Solid lines indicate ORs and shadow shapes indicate 95% Cis. Graphs show ORs for MASLD adjusted for age, gender, race/ethnicity, PIR, education, marital status, health insurance, tobacco use, alcohol use, hypertension, diabetes, cardiovascular disease, WC, PA, BMI, TG, HDL, ALT, AST, GGT.

The Restricted Cubic Spline (RCS) analysis, illustrated in Figure 2, demonstrated a linear dose–response relationship between SII, SIRI, LMR, NLR, PLR, and NPAR and MASLD (*p* for nonlinear >0.05), with no significant nonlinear turning point observed. We observed that the risk of MASLD increased with increasing scores of SII, SIRI, and NPAR (*p* for overall <0.05). However, a significant dose–response association between LMR, NLR, and PLR scores and MASLD was not evident (*p* for overall >0.05).

### 3.3 Logistic regression analysis associations between systemic immune biomarkers and MASLD

**Figure 3:**
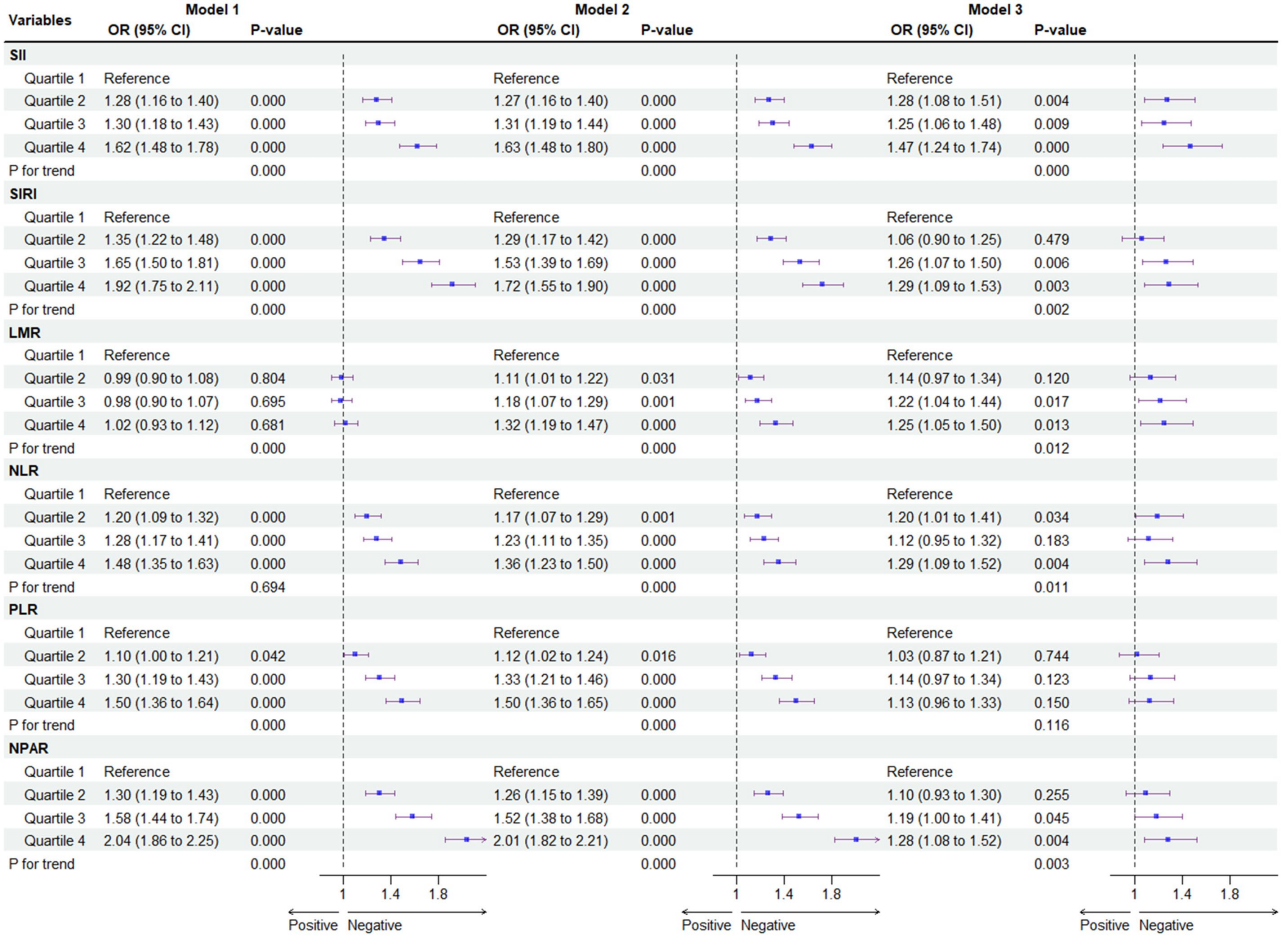
Association between systemic immune biomarkers and MASLD. For SII, Median [Range]: Quartiles 1, 244.38 [1.53 to 313.50]; Quartiles 2, 374.29 [313.51 to 440.00]; Quartiles 3, 520.00 [440.01 to 625.73]; Quartiles 4, 804.15 [625.74 to 28397.28]; For SIRI, Median [Range]: Quartiles 1, 0.48 [0.06 to 0.64]; Quartiles 2, 0.80 [0.65 to 0.95]; Quartiles 3, 1.14 [0.96 to 1.42]; Quartiles 4, 1.91 [1.43 to 22.92]; For LMR, Median [Range]: Quartiles 1, 2.50 [0.44 to 3.00]; Quartiles 2, 3.50 [3.01 to 3.86]; Quartiles 3, 4.40 [3.87 to 5.00]; Quartiles 4, 6.00 [5.01 to 55.4]; For NLR, Median [Range]: Quartiles 1, 1.14 [0.01 to 1.42]; Quartiles 2, 1.65 [1.43 to 1.89]; Quartiles 3, 2.17 [1.90 to 2.54]; Quartiles 4, 3.17 [2.55 to 28.66]; For PLR, Median [Range]: Quartiles 1, 0.006 [0.001 to 0.007]; Quartiles 2, 0.008 [0.007 to 0.008]; Quartiles 3, 0.009 [0.008 to 0.011]; Quartiles 4, 0.012 [0.011 to 1.036]; For NPAR, Median [Range]: Quartiles 1, 1.08 [0.02 to 1.20]; Quartiles 2, 1.28 [1.21 to 1.36]; Quartiles 3, 1.45 [1.37 to 1.54]; Quartiles 4, 1.67 [1.55 to 3.03] *p < 0.05 is considered statistically significant. Model 1 was the unadjusted model. Model 2 was adjusted for gender, age, race/ethnicity, PIR, education, marital status, health insurance. Model 3 was further adjusted for tobacco use, alcohol use, hypertension, T2DM, cardiovascular disease, WC, PA, body mass index (BMI), TG, HDL, ALT, AST, and GGT.

Figure 3 depicts the correlation between different systemic immune biomarkers and the prevalence of MASLD. Our findings indicate that higher SII, SIRI, LMR, NLR, and NPAR are associated with an increased risk of MASLD, regardless of adjustments for confounding factors. In the crude model, NPAR exhibited the highest odds ratio (OR) per standard deviation change among the six systemic immune biomarkers [OR = 2.04; 95% CI (1.86, 2.24), Q4 of NPAR vs. Q1]. After fully adjusting for potential confounders, SII [OR = 1.47; 95% CI (1.24, 1.74), Q4 of SII vs. Q1] was associated with the highest OR per standard deviation increment. Multicollinearity was not present for all variables (variance inflation factor, VIF < 5), as shown in Supplementary Table S1.

**Figure 4:**
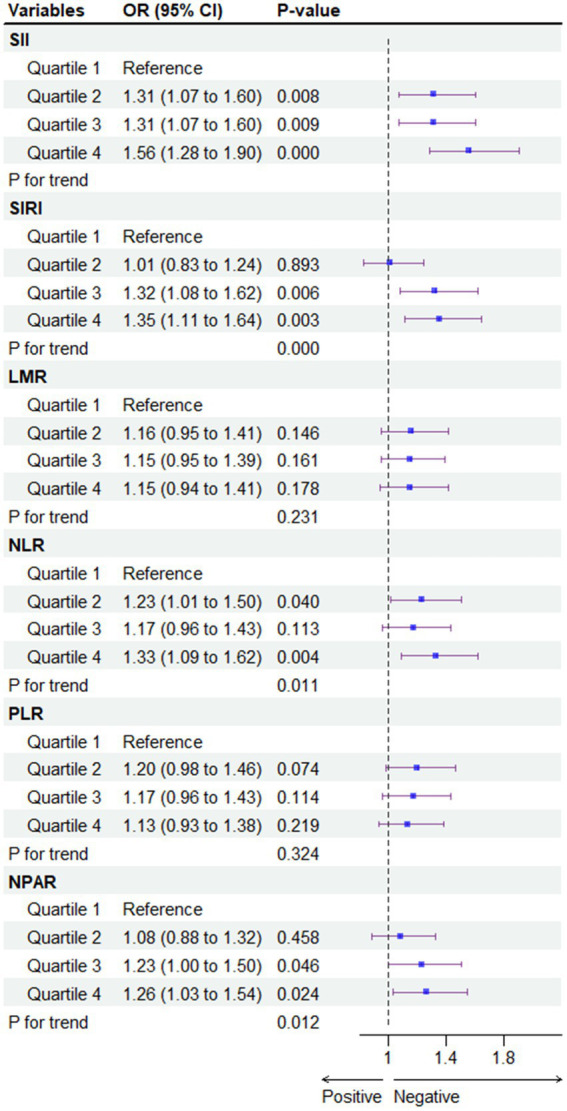
Association between systemic immune biomarkers and MASLD after propensity score matching analysis. CDAI, composite dietary antioxidant index; Q, quartile. *p < 0.05 is considered statistically significant. One-to-one matching between MASLD and non-MASLD was conducted based on age, gender, race/ethnicity, poverty income ratio (PIR), education, marital status, health insurance, tobacco use, alcohol use, hypertension, diabetes, cardiovascular disease, WC, PA, BMI, TG, HDL, ALT, AST, GGT.

To mitigate potential selection bias due to potential outcomes, a 1:1 match was conducted using propensity score matching (PSM), resulting in the matching of 1,570 participants with non-MASLD and 1,570 participants with MASLD (Figure 4). Compared to the first quartile (Q1), the association of the Q4 of SII [OR = 1.62; 95% CI (1.48, 1.78)], SIRI [OR = 1.92; 95% CI (1.75, 2.11)], NLR [OR = 1.48; 95% CI (1.35, 1.63)], and NPAR [OR = 2.04; 95% CI (1.86, 2.24)] with the risk of developing MASLD persisted in the cohort matched for propensity scores. However, this association for the full quartile of LMR and PLR was not observed (*p* > 0.05). All statistical results of the logistic regression model exploring the association between systemic immune biomarkers and MASLD and MASLD-related fibrosis are presented in Supplementary Tables S2–S6.

### 3.4 ROC analysis of the predictive value of systemic immune biomarkers for MASLD

Table 2 presents the ROC curves for identifying participants with MASLD. The optimal cut-off values, determined using the Youden index via the receiver operating characteristic curve (ROC), were as follows: 339.27 for SII, 0.863 for SIRI, 5.354 for LMR, 1.649 for NLR, 0.009 for PLR, and 1.36 for NPAR. NPAR exhibited the highest discrimination ability [AUC = 0.711; 95% CI (0.702, 0.719), *p* for DeLong’s test <0.05, *p* for Hosmer–Lemeshow test >0.05] among all six systemic immune biomarkers. Additionally, the AUCs for SII, SIRI, LMR, NLR, and PLR were 0.707 [95% CI (0.698, 0.715)], 0.707 [95% CI (0.698, 0.715)], 0.705 [95% CI (0.696, 0.713)], 0.704 [95% CI (0.696, 0.713)], and 0.705 [95% CI (0.697, 0.714)], respectively.

**Table 2:** ROC curves of different systemic immune biomarkers for MASLD.

Variable	Sensitivity^a^	Specificity^a^	AUC (95%)	*p*-value^b^	Standard error^c^	*p* for HL test^d^
**Model 1**						
SII	0.737	0.334	0.549 (0.539,0.558)	0.000	0.005	0.434
SIRI	0.626	0.483	0.571 (0.561,0.580)	0.000	0.005	0.000
LMR	0.189	0.821	0.501 (0.492,0.511)	0.792	0.005	0.298
NLR	0.665	0.404	0.542 (0.532,0.551)	0.000	0.005	0.000
PLR	0.513	0.562	0.546 (0.537,0.556)	0.000	0.005	0.000
NPAR	0.571	0.546	0.580 (0.570,0.589)	0.000	0.005	0.917
**Model 2**						
SII	0.655	0.654	0.707 (0.698,0.715)	0.000	0.004	0.226
SIRI	0.662	0.642	0.707 (0.698,0.715)	0.000	0.004	0.357
LMR	0.717	0.59	0.705 (0.696,0.713)	0.000	0.004	0.091
NLR	0.711	0.592	0.704 (0.696,0.713)	0.000	0.004	0.170
PLR	0.722	0.533	0.705 (0.697,0.714)	0.000	0.004	0.057
NPAR	0.627	0.546	0.711 (0.702,0.719)	0.000	0.004	0.434

AUC, area under curve; ROC, receiver operating characteristics curve. Model 1 was the unadjusted model. Model 2 was adjusted for gender, age, race/ethnicity, PIR, education, marital status, health insurance, tobacco use, alcohol use, hypertension, diabetes, cardiovascular disease.

a Sensitivity and specificity were calculated using the best thresholds according to Youden’s index.

b Assume nonparametric.

c Null hypothesis: True region = 0.5.

d Hosmer–Lemeshow test.

### 3.5 Subgroup analysis between systemic immune biomarkers and MASLD

**Figure 5:**
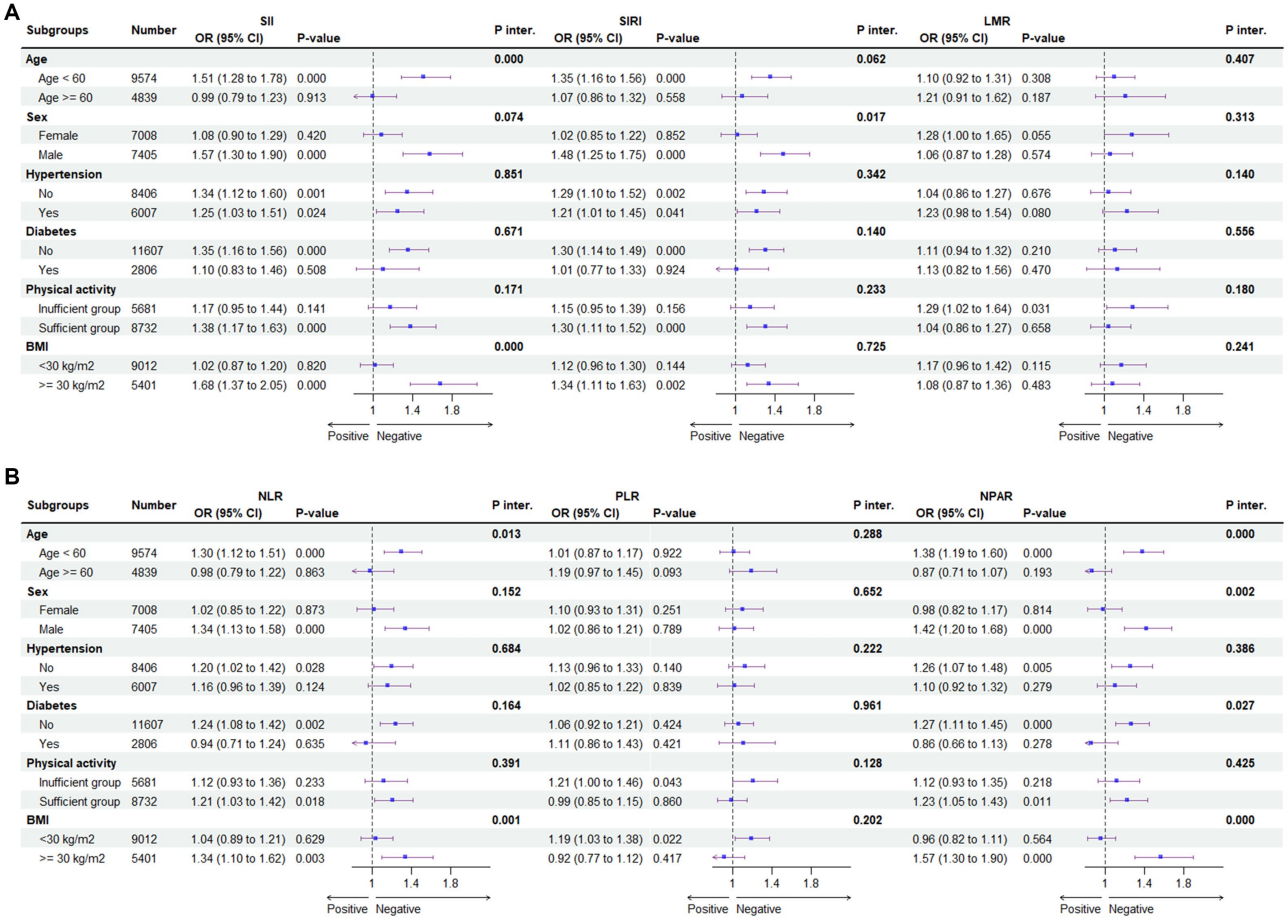
Subgroup analysis for the association between SII, SIRI, LMR **(A)** and NLR, PLR, NPAR **(B)** and the risk of MASLD. BMI, body mass index; CDAI, composite dietary antioxidant index; Q, quartile *p < 0.05 is considered statistically significant. According to the Youden index, the receiver operating characteristic curve (ROC) was used to determine the optimal cut-off values for SII, SIRI, LMR, NLR, PLR, and NPAR were 339.27, 0.863, 5.354, 1.649, 0.009, and 1.36, respectively. ORs were fully adjusted by the following covariates including age, gender, race/ethnicity, PIR, education, marital status, health insurance, tobacco use, alcohol use, hypertension, diabetes, cardiovascular disease, WC, PA, body mass index (BMI), TG, HDL, ALT, AST, GGT.

Figure 5 shows the subgroup analysis of the association between systemic immune biomarkers and the risk of MASLD. Our research revealed that there was no dependence on the association between SIRI, LMR, PLR and the risk of MASLD. The interaction test revealed no significant differences in terms of age, sex, hypertension, diabetes, physical activity, and BMI in the association between the above three immune biomarkers and MASLD, indicating that these factors had no significant influence on this positive relationship (all *p* for interaction >0.05). However, age and BMI have a significant interaction effect on the association between SII, NLR, NPAR and MASLD (all *p* for interaction <0.05). A significant association of SII, NLR, and NPAR with MASLD risk was revealed in the subgroup with age < 60 and BMI > 30. However, after Bonferroni correction, the association of SII, NLR, and NPAR in the subgroup of age- and BMI-based controls and MASLD risk persisted significantly, while no other associations were found.

## 4 Discussion

### 4.1 Main finding

The escalating incidence of MASLD is a consequence of evolving lifestyles, dietary patterns, diminished physical activity, and the accelerated pace of contemporary living, emerging as a global public health concern (54). The prevalence of MASLD within each country’s general population ranges from 10 to 24%, with a notably higher prevalence in women compared to men. In the United States, the estimated prevalence of MASLD is between 16 and 23% (6). Given the substantial population afflicted by MASLD, there is an imperative to prioritize the early detection and treatment of this disease.

In this comprehensive study, which scrutinized the most representative U.S. population data, our analysis revealed a positive correlation between the prevalence of MASLD and all six systemic immune biomarkers (SII, SIRI, LMR, NLR, PLR, and NPAR). However, following propensity matching, this association remained significant only for SII, SIRI, NLR, and NPAR. Moreover, the Restricted Cubic Spline (RCS) analysis unveiled that SII, SIRI, and NPAR exhibited a linear relationship with the risk of MASLD, signifying a proportional increase in MASLD risk with their elevation. Notably, NPAR demonstrated the satisfactory predictive value, as substantiated by the results of Receiver Operating Characteristic (ROC) analysis and subgroup analysis. Additionally, SIRI and SII exhibited comparable predictive values to NPAR while maintaining the advantage of simpler calculations. These findings underscore the nuanced associations between systemic immune biomarkers and MASLD prevalence, offering valuable insights into the predictive capacities of individual biomarkers.

### 4.2 Comparison with other studies

SII, SIRI, LMR, NLR, PLR, and NPAR serve as effective immune biomarkers of immune and inflammatory status in the human body, and previous epidemiological studies and meta-analyses have demonstrated a correlation between these inflammatory indices and liver disease. In a Chinese population cohort of 376 patients with decompensated cirrhosis, higher NPAR was independently associated with an increased risk of death in patients with cirrhosis after adjusting for confounders [HR_Q3vs.Q1_ = 1.92; 95% CI (1.04, 3.56)], with a one-unit increase in NPAR associated with a 92% increased risk of mortality (55). In a cross-sectional analysis of 2017–2018 NHANES data, Liu et al. (22) found that increasing NLR and NPAR were significantly linked to a higher risk of developing MASLD. Both NLR and NPAR were also associated with an increased likelihood of advanced fibrosis. The novel biomarker NPAR demonstrated a strong association with MASLD in the national population and participants’ clinical characteristics. NPAR can serve as a valuable biomarker for MASLD, aiding clinicians in enhancing the diagnosis and treatment of chronic liver disease. Zhao et al. (56) found that higher SII levels are associated with increased mortality in ultrasound-diagnosed MASLD populations. The study reveals a J-shaped curve in SII and all-cause death within the MASLD group. These results suggest that SII could serve as a useful prognostic indicator for all-cause mortality in MASLD, and maintaining SII levels below a certain threshold may help reduce the risk of death. Future research should aim for a deeper understanding of how interfering with SII impacts the survival of those with MASLD. LMR reflects the equilibrium between anti-tumor immune response and tumor-promoting functions. It holds prognostic significance as an indicator of inflammatory response in hepatocellular carcinoma. Notably, a statistically significant association exists between low LMR expression and hepatocellular carcinoma in patients (57). When investigating the relationship between different immune markers and MASLD, we observed some significant differences. Our results show that there is no significant correlation between PLR and the risk of MASLD. However, a study showed that the incidence of MASLD was significantly reduced. This difference may be due to different confounding factors, different diagnostic criteria of MASLD, and different sample sizes. To further explore this phenomenon, we suggest that future research should be carried out under a larger sample size and a more unified diagnostic standard, and the potential confounding factors should be adjusted more comprehensively. In this way, more robust and accurate results can be obtained, and the relationship between PLR and MASLD risk can be better understood.

MASLD is the most common steatosis of the liver, which induces oxidative stress and inflammation due to lipid accumulation in liver cells and may eventually develop into cirrhosis. In the case of lipid overload, the activation and recruitment of liver immune cells produce inflammatory cytokines and chemokines such as tumor necrosis factor-α (TNF-α), interleukin-1β (IL-1β), and interleukin-6 (IL-6), which further enhance the inflammatory response and lead to liver cell injury and inflammatory necrosis (58). The immuno-inflammatory index integrates various types of inflammatory cells, including platelets, neutrophils, and lymphocytes, and objectively reflects the balance between inflammatory and immune responses. Our study revealed that higher SII, SIRI, NLR, and NPLR are independently associated with an increased risk of hepatic steatosis. This suggests that these inflammatory indices may have significant adverse effects on hepatic steatosis independently. Currently, there are no specifically approved drugs for the treatment of MASLD, so lifestyle changes and managing risk factors are the primary treatments for people with MASLD (2). A high-salt diet (HSD), defined as an intake of more than 5 grams of salt per day (59), has been shown to trigger a systemic inflammatory response. This inflammatory response weakens the immune system by stimulating pro-inflammatory T cells (60) and suppressing natural killer cells (61). Long-term HSD is associated with a variety of diseases, including cardiovascular disease, cancer, chronic inflammation, and autoimmune diseases (61). Therefore, given the relationship between HSD and the immune inflammatory state of the body, it may be possible to prevent or improve the progression of MASLD through anti-inflammatory diets (such as low-salt diets) in the future. We expect that future studies will further validate these findings and provide support for more specific interventions.

### 4.3 Strengths and limitations

The study comprehensively explored the direct relationship between six systemic immune biomarkers (SII, SIRI, NLR, PLR, and NPAR) and MASLD, providing valuable insights to improve understanding of MASLD and inflammation and develop more effective medical strategies. Additionally, this study utilized NHANES data from 2007 to 2018, covering six survey cycles, providing a wide period, sufficient sample size, and representative samples. However, there are some limitations to this study. Firstly, the cross-sectional observational design limits its ability to make definitive causal conclusions due to the lack of information on the timing of the association. Secondly, Residual confounding by unmeasured covariates cannot be disregarded entirely which could affect our observed results and introduce some bias. Thirdly, given that a significant number of subjects with missing liver and complete blood cell data from NHANES were excluded from our observational analysis, potential selection bias might have been introduced. Finally, the absence of a gold standard for diagnosing MASLD or NASH may introduce diagnostic bias. Therefore, further large-scale cohort studies are necessary to elucidate the intricate relationship between systemic immune biomarkers and MASLD.

## 5 Conclusion

The study investigated the link between systemic immune biomarkers and MASLD in a representative U.S. adult sample. Results revealed a significant positive correlation between elevated levels of SII, SIRI, NLR, and NPAR with a heightened risk of MASLD. These associations remained robust in sensitivity analysis. SII, SIRI, NLR, and NPAR may serve as a biomarker for MASLD and help clinicians refine the diagnosis and treatment of chronic liver disease, where NPAR showed the superior predictive value. The results of this study could reveal the potential role of inflammation in MASLD, it may be possible to prevent or improve the progression of MASLD through anti-inflammatory diets, such as low-salt diets. Longitudinal studies and clinical trials are needed to further characterize and confirm the findings presented herein.

## Data Availability

The raw data supporting the conclusions of this article will be made available by the authors, without undue reservation.

## Footnotes

^1^ https://www.cdc.gov/nchs/nhanes/index.htm

## References

[1] Chalasani N Younossi Z Lavine JE Charlton M Cusi K Rinella M. The diagnosis and management of nonalcoholic fatty liver disease: practice guidance from the American Association for the Study of Liver Diseases. Hepatology. (2018) 67:328–57. doi: 10.1002/hep.29367, PMID: 28714183

[2] Lazarus JV Mark HE Anstee QM Arab JP Batterham RL Castera L. Advancing the global public health agenda for NAFLD: a consensus statement. Nat Rev Gastroenterol Hepatol. (2022) 19:60–78. doi: 10.1038/s41575-021-00523-4, PMID: 34707258

[3] Targher G Byrne CD Tilg H. NAFLD and increased risk of cardiovascular disease: clinical associations, pathophysiological mechanisms and pharmacological implications. Gut. (2020) 69:1691–705. doi: 10.1136/gutjnl-2020-320622, PMID: 32321858

[4] Song Y Guo W Li Z Guo D Li Z Li Y. Systemic immune-inflammation index is associated with hepatic steatosis: Evidence from NHANES 2015-2018. Front Immunol. (2022) 13:1058779. doi: 10.3389/fimmu.2022.1058779, PMID: 36466832 PMC9718528

[5] Le MH Yeo YH Li X Li J Zou B Wu Y. 2019 Global NAFLD Prevalence: a systematic review and meta-analysis. Clin Gastroenterol Hepatol. (2022) 20:2809–2817.e28. doi: 10.1016/j.cgh.2021.12.002, PMID: 34890795

[6] Estes C Anstee QM Arias-Loste MT Bantel H Bellentani S Caballeria J. Modeling NAFLD disease burden in China, France, Germany, Italy, Japan, Spain, United Kingdom, and United States for the period 2016–2030. J Hepatol. (2018) 69:896–904. doi: 10.1016/j.jhep.2018.05.036, PMID: 29886156

[7] Younossi Z Anstee QM Marietti M Hardy T Henry L Eslam M. Global burden of NAFLD and NASH: trends, predictions, risk factors and prevention. Nat Rev Gastroenterol Hepatol. (2018) 15:11–20. doi: 10.1038/nrgastro.2017.109 28930295

[8] Song Y Zhang J Wang H Guo D Yuan C Liu B. A novel immune-related genes signature after bariatric surgery is histologically associated with non-alcoholic fatty liver disease. Adipocytes. (2021) 10:424–34. doi: 10.1080/21623945.2021.1970341, PMID: 34506234 PMC8437528

[9] Koyama Y Brenner DA. Liver inflammation and fibrosis. J Clin Invest. (2017) 127:55–64. doi: 10.1172/jci88881 28045404 PMC5199698

[10] du Plessis J van Pelt J Korf H Mathieu C van der Schueren B Lannoo M. Association of adipose tissue inflammation with histologic severity of nonalcoholic fatty liver disease. Gastroenterology. (2015) 149:635–48.e14. doi: 10.1053/j.gastro.2015.05.044, PMID: 26028579

[11] Csak T Ganz M Pespisa J Kodys K Dolganiuc A Szabo G. Fatty acid and endotoxin activate inflammasomes in mouse hepatocytes that release danger signals to stimulate immune cells. Hepatology. (2011) 54:133–44. doi: 10.1002/hep.24341, PMID: 21488066 PMC4158408

[12] Biyik M Ucar R Solak Y Gungor G Polat I Gaipov A. Blood neutrophil-to-lymphocyte ratio independently predicts survival in patients with liver cirrhosis. Eur J Gastroenterol Hepatol. (2013) 25:435–41. doi: 10.1097/MEG.0b013e32835c2af3 23249602

[13] Liu C Gao Y Ji J Sun C Chen M. Association between inflammatory indexes and erectile dysfunction in U.S. adults: National Health and Nutrition Examination Survey 2001–2004. Sex Med. (2023) 11:e45. doi: 10.1093/sexmed/qfad045, PMID: 37577069 PMC10413424

[14] Zhao S Dong S Qin Y Wang Y Zhang B Liu A. Inflammation index SIRI is associated with increased all-cause and cardiovascular mortality among patients with hypertension. Front Cardiovasc Med. (2022) 9:1066219. doi: 10.3389/fcvm.2022.1066219, PMID: 36712259 PMC9874155

[15] Wenpei G Yuan L Liangbo L Jingjun M Bo W Zhiqiang N. Predictive value of preoperative inflammatory indexes for postoperative early recurrence of hepatitis B-related hepatocellular carcinoma. Front Oncol. (2023) 13:1142168. doi: 10.3389/fonc.2023.1142168, PMID: 37519805 PMC10373589

[16] Cui S Cao S Chen Q He Q Lang R. Preoperative systemic inflammatory response index predicts the prognosis of patients with hepatocellular carcinoma after liver transplantation. Front Immunol. (2023) 14:1118053. doi: 10.3389/fimmu.2023.1118053 37051235 PMC10083266

[17] Lobo PCB de Branco FMS Pichard C de Oliveira EP Pimentel GD. C-reactive protein, but not neutrophil-lymphocyte ratio, is inversely associated with muscle strength only in older men: NHANES 1999–2002. Exp Gerontol. (2023) 173:112084. doi: 10.1016/j.exger.2023.112084 36634720

[18] Dong G Gan M Xu S Xie Y Zhou M Wu L. The neutrophil-lymphocyte ratio as a risk factor for all-cause and cardiovascular mortality among individuals with diabetes: evidence from the NHANES 2003–2016. Cardiovasc Diabetol. (2023) 22:267. doi: 10.1186/s12933-023-01998-y, PMID: 37775767 PMC10541705

[19] Zhou D Wang J Li X. The Platelet-Lymphocyte Ratio Associated with Depression in Diabetes Patients in the US National Health and Nutrition Examination Survey. Int J Gen Med. (2021) 14:7825–32. doi: 10.2147/ijgm.S334883, PMID: 34795503 PMC8593353

[20] Wu CC Wu CH Lee CH Cheng CI. Association between neutrophil percentage-to-albumin ratio (NPAR), neutrophil-to-lymphocyte ratio (NLR), platelet-to-lymphocyte ratio (PLR) and long-term mortality in community-dwelling adults with heart failure: evidence from US NHANES 2005–2016. BMC Cardiovasc Disord. (2023) 23:312. doi: 10.1186/s12872-023-03316-6, PMID: 37344786 PMC10286403

[21] Zhao B Liu Y Yang Y He J. Association of systemic immune-inflammation index with non-alcoholic fatty liver disease: a population-based cross-sectional study. Risk Manag Healthc Policy. (2023) 16:1581–92. doi: 10.2147/rmhp.S419183, PMID: 37605743 PMC10440121

[22] Liu CF Chien LW. Predictive role of neutrophil-percentage-to-albumin ratio (NPAR) in nonalcoholic fatty liver disease and advanced liver fibrosis in nondiabetic US adults: evidence from NHANES 2017–2018. Nutrients. (2023) 15:81892. doi: 10.3390/nu15081892 PMC10141547 37111111

[23] Ghaferi AA Schwartz TA Pawlik TM. STROBE reporting guidelines for observational studies. JAMA Surg. (2021) 156:577–8. doi: 10.1001/jamasurg.2021.0528 33825815

[24] Bedogni G Bellentani S Miglioli L Masutti F Passalacqua M Castiglione A. The fatty liver index: a simple and accurate predictor of hepatic steatosis in the general population. BMC Gastroenterol. (2006) 6:33. doi: 10.1186/1471-230x-6-33, PMID: 17081293 PMC1636651

[25] Ruhl CE Everhart JE. Fatty liver indices in the multiethnic United States National Health and Nutrition Examination Survey. Aliment Pharmacol Ther. (2015) 41:65–76. doi: 10.1111/apt.13012 25376360

[26] Li L Huang Q Yang L Zhang R Gao L Han X. The Association between Non-Alcoholic Fatty Liver Disease (NAFLD) and Advanced Fibrosis with Serological Vitamin B12 Markers: Results from the NHANES 1999–2004. Nutrients. (2022) 14:61224. doi: 10.3390/nu14061224, PMID: 35334881 PMC8948655

[27] Sterling RK Lissen E Clumeck N Sola R Correa MC Montaner J. Development of a simple noninvasive index to predict significant fibrosis in patients with HIV/HCV coinfection. Hepatology. (2006) 43:1317–25. doi: 10.1002/hep.21178, PMID: 16729309

[28] Angulo P Hui JM Marchesini G Bugianesi E George J Farrell GC. The NAFLD fibrosis score: a noninvasive system that identifies liver fibrosis in patients with NAFLD. Hepatology. (2007) 45:846–54. doi: 10.1002/hep.21496, PMID: 17393509

[29] Cheah MC McCullough AJ Goh GB. Current modalities of fibrosis assessment in non-alcoholic fatty liver disease. J Clin Transl Hepatol. (2017) 2017:1–11. doi: 10.14218/jcth.2017.00009, PMID: 28936407 PMC5606972

[30] Gangrade N Figueroa J Leak TM. Socioeconomic Disparities in Foods/Beverages and Nutrients Consumed by U.S. Adolescents When Snacking: National Health and Nutrition Examination Survey 2005–2018. Nutrients. (2021) 13:82530. doi: 10.3390/nu13082530 PMC8399168 34444690

[31] American Diabetes Association Professional Practice Committee. 2. Classification and Diagnosis of Diabetes: Standards of Medical Care in Diabetes-2022. Diabetes Care. (2022) 45:S17–38. doi: 10.2337/dc22-S002, PMID: 34964875

[32] Siegel LN Cook S Oh H Liber AC Levy DT Fleischer NL. The longitudinal association between coupon receipt and established cigarette smoking initiation among young adults in USA. Tob Control. (2023):tc-2023-058065. doi: 10.1136/tc-2023-058065, PMID: 37468154 PMC10796848

[33] Lawrence DM Hunt A Mathews B Haslam DM Malacova E Dunne MP. The association between child maltreatment and health risk behaviours and conditions throughout life in the Australian Child Maltreatment Study. Med J Aust. (2023) 218:S34–9. doi: 10.5694/mja2.51877, PMID: 37004181 PMC10952518

[34] Chen R Wang K Chen Q Zhang M Yang H Zhang M. Weekend warrior physical activity pattern is associated with lower depression risk: Findings from NHANES 2007–2018. Gen Hosp Psychiatry. (2023) 84:165–71. doi: 10.1016/j.genhosppsych.2023.07.006, PMID: 37535993

[35] Han M Fang J Zhang Y Song X Jin L Ma Y. Associations of sleeping, sedentary and physical activity with phenotypic age acceleration: a cross-sectional isotemporal substitution model. BMC Geriatr. (2023) 23:165. doi: 10.1186/s12877-023-03874-6, PMID: 36959562 PMC10035275

[36] Piercy KL Troiano RP Ballard RM Carlson SA Fulton JE Galuska DA. The Physical Activity Guidelines for Americans. JAMA. (2018) 320:2020–8. doi: 10.1001/jama.2018.14854, PMID: 30418471 PMC9582631

[37] Cao C Friedenreich CM Yang L. Association of daily sitting time and leisure-time physical activity with survival among us cancer survivors. JAMA Oncol. (2022) 8:395–403. doi: 10.1001/jamaoncol.2021.6590, PMID: 34989765 PMC8739832

[38] Durrleman S Simon R. Flexible regression models with cubic splines. Stat Med. (1989) 8:551–61. doi: 10.1002/sim.4780080504 2657958

[39] Harrell FE. Regression modeling strategies: with applications to linear models, logistic regression, and survival analysis Springer (2015).

[40] Qi G Wang J Chen Y Wei W Sun C. Association between dietary spermidine intake and depressive symptoms among US adults: National Health and Nutrition Examination Survey (NHANES) 2005-2014. J Affect Disord. (2024) 359:125–32. doi: 10.1016/j.jad.2024.05.041, PMID: 38729223

[41] Jiang H Yang G Chen J Yuan S Wu J Zhang J. The correlation between selenium intake and lung function in asthmatic people: a cross-sectional study. Front Nutr. (2024) 11:1362119. doi: 10.3389/fnut.2024.1362119, PMID: 38826577 PMC11141543

[42] Schafer JL. Multiple imputation: a primer. Stat Methods Med Res. (1999) 8:3–15. doi: 10.1177/096228029900800102, PMID: 10347857

[43] White IR Royston P Wood AM. Multiple imputation using chained equations: Issues and guidance for practice. Stat Med. (2011) 30:377–99. doi: 10.1002/sim.4067, PMID: 21225900

[44] Mul Fedele ML López Gabeiras MDP Simonelli G Diez JJ Bellone GJ Cagliani J. Multivariate analysis of the impact of sleep and working hours on medical errors: a MICE approach. BMC Public Health. (2023) 23:2317. doi: 10.1186/s12889-023-17130-4, PMID: 37996804 PMC10666331

[45] Kilpeläinen TP Tikkinen KAO Guyatt GH Vernooij RWM. Evidence-based urology: subgroup analysis in randomized controlled trials. Eur Urol Focus. (2021) 7:1237–9. doi: 10.1016/j.euf.2021.10.001, PMID: 34688589

[46] Benjamini Y Drai D Elmer G Kafkafi N Golani I. Controlling the false discovery rate in behavior genetics research. Behav Brain Res. (2001) 125:279–84. doi: 10.1016/s0166-4328(01)00297-2 11682119

[47] Jiang H Fan Y Li J Wang J Kong L Wang L. The associations of plasma carotenoids and vitamins with risk of age-related macular degeneration: results from a matched case-control study in china and meta-analysis. Front Nutr. (2022) 9:745390. doi: 10.3389/fnut.2022.745390, PMID: 35223939 PMC8873933

[48] Logue Cook RN Brown SH Hasson RE Kinnett-Hopkins D Davis MA. Is maximum grip strength a reliable predictor of hand limitations among older adults? Aging Clin Exp Res. (2022) 34:2505–14. doi: 10.1007/s40520-022-02191-z, PMID: 35871136

[49] Swets JA. Measuring the accuracy of diagnostic systems. Science. (1988) 240:1285–93. doi: 10.1126/science.3287615 3287615

[50] Hosmer DW Lemesbow S. Goodness of fit tests for the multiple logistic regression model. Commun Stat Theory Methods. (1980) 9:1043–69. doi: 10.1080/03610928008827941

[51] Nicoară-Farcău O Wang X Luo X. Definition of SPSS: we need to speak the same language. J Hepatol. (2020) 73:463–4. doi: 10.1016/j.jhep.2020.03.012, PMID: 32448470

[52] Shim SR Kim SJ. Intervention meta-analysis: application and practice using R software. Epidemiol Health. (2019) 41:e2019008. doi: 10.4178/epih.e2019008, PMID: 30999738 PMC6545497

[53] Wang X Seo YA Park SK. Serum selenium and non-alcoholic fatty liver disease (NAFLD) in U.S. adults: National Health and Nutrition Examination Survey (NHANES) 2011-2016. Environ Res. (2021) 197:111190. doi: 10.1016/j.envres.2021.111190 33872646 PMC8187321

[54] Younossi ZM. Non-alcoholic fatty liver disease—a global public health perspective. J Hepatol. (2019) 70:531–44. doi: 10.1016/j.jhep.2018.10.033 30414863

[55] Du X Wei X Ma L Liu X Guo H Liu Y. Higher levels of neutrophil percentage-to-albumin ratio predict increased mortality risk in patients with liver cirrhosis: a retrospective cohort study. Eur J Gastroenterol Hepatol. (2023) 35:198–203. doi: 10.1097/meg.0000000000002470, PMID: 36472501 PMC9770107

[56] Zhao E Cheng Y Yu C Li H Fan X. The systemic immune-inflammation index was non-linear associated with all-cause mortality in individuals with nonalcoholic fatty liver disease. Ann Med. (2023) 55:2197652. doi: 10.1080/07853890.2023.2197652, PMID: 37052341 PMC10115001

[57] Itoh S Yugawa K Shimokawa M Yoshiya S Mano Y Takeishi K. Prognostic significance of inflammatory biomarkers in hepatocellular carcinoma following hepatic resection. BJS Open. (2019) 3:500–8. doi: 10.1002/bjs5.50170, PMID: 31388642 PMC6677099

[58] Peverill W Powell LW Skoien R. Evolving concepts in the pathogenesis of NASH: beyond steatosis and inflammation. Int J Mol Sci. (2014) 15:8591–638. doi: 10.3390/ijms15058591, PMID: 24830559 PMC4057750

[59] Brown IJ Tzoulaki I Candeias V Elliott P. Salt intakes around the world: implications for public health. Int J Epidemiol. (2009) 38:791–813. doi: 10.1093/ije/dyp139, PMID: 19351697

[60] Musiol S Harris CP Gschwendtner S Burrell A Amar Y Schnautz B. The impact of high-salt diet on asthma in humans and mice: Effect on specific T-cell signatures and microbiome. Allergy. (2024) 79:1844–57. doi: 10.1111/all.16148, PMID: 38798015

[61] Zeng X Li Y Lv W Dong X Zeng C Zeng L. A high-salt diet disturbs the development and function of natural killer cells in mice. J Immunol Res. (2020) 2020:6687143. doi: 10.1155/2020/6687143, PMID: 33426093 PMC7772026

