# Supplementary material for: Association of systemic immune biomarkers with metabolic dysfunction-associated steatotic liver disease: a cross-sectional study of NHANES 2007–2018
Source: Front Nutr. 2024 Sep 4;11:1415484. doi: 10.3389/fnut.2024.1415484 (PMC11408230; doi:10.3389/fnut.2024.1415484)
Supplement: Supplementary file 1 [file Table_1.DOCX]

Supplementary Material

| **TABLE S1** Determination of coefficients and variance inflation factor of the variables | | | | | | | | | |
| --- | --- | --- | --- | --- | --- | --- | --- | --- | --- |
| Model | Unstandardized Coefficients | | Standardized Coefficients | t | Sig. | 95.0% Confidence Interval for B | | Collinearity Statistics | |
|  | B | Standard Error | Beta |  |  | Lower Bound | Upper Bound | Tolerance | VIF |
| (Constant) | -0.238 | 0.027 |  | -8.822 | 0.000 | -0.291 | -0.185 |  |  |
| Age | 0.000 | 0.000 | -0.007 | -1.068 | 0.285 | -0.001 | 0.000 | 0.592 | 1.689 |
| Gender | -0.083 | 0.006 | -0.084 | -13.552 | 0.000 | -0.096 | -0.071 | 0.761 | 1.314 |
| Race/Ethnicity | -0.008 | 0.002 | -0.020 | -3.407 | 0.001 | -0.013 | -0.004 | 0.867 | 1.153 |
| PIR | 0.001 | 0.006 | 0.001 | 0.141 | 0.888 | -0.011 | 0.013 | 0.842 | 1.187 |
| Education | 0.004 | 0.007 | 0.003 | 0.575 | 0.565 | -0.009 | 0.017 | 0.828 | 1.208 |
| Marital status | -0.004 | 0.004 | -0.006 | -0.986 | 0.324 | -0.011 | 0.004 | 0.908 | 1.101 |
| Health insurance | 0.007 | 0.007 | 0.006 | 0.977 | 0.329 | -0.007 | 0.021 | 0.833 | 1.201 |
| Alcohol use | 0.001 | 0.006 | 0.001 | 0.094 | 0.925 | -0.011 | 0.012 | 0.905 | 1.105 |
| Tobacco use | -0.016 | 0.006 | -0.016 | -2.752 | 0.006 | -0.027 | -0.005 | 0.881 | 1.136 |
| Hypertension | 0.028 | 0.006 | 0.028 | 4.346 | 0.000 | 0.015 | 0.041 | 0.712 | 1.404 |
| Diabetes | 0.094 | 0.007 | 0.075 | 12.601 | 0.000 | 0.079 | 0.108 | 0.833 | 1.200 |
| Cardiovascular diseases | 0.009 | 0.006 | 0.008 | 1.356 | 0.175 | -0.004 | 0.021 | 0.926 | 1.080 |
| WC | 0.142 | 0.008 | 0.141 | 16.734 | 0.000 | 0.125 | 0.159 | 0.409 | 2.448 |
| Physical activity | -0.025 | 0.006 | -0.024 | -4.257 | 0.000 | -0.036 | -0.013 | 0.907 | 1.103 |
| BMI | 0.315 | 0.005 | 0.515 | 63.028 | 0.000 | 0.306 | 0.325 | 0.436 | 2.294 |
| TG | 0.216 | 0.007 | 0.177 | 31.491 | 0.000 | 0.203 | 0.229 | 0.926 | 1.080 |
| HDL | -0.046 | 0.016 | -0.016 | -2.853 | 0.004 | -0.078 | -0.015 | 0.952 | 1.050 |
| ALT | 0.003 | 0.000 | 0.110 | 12.254 | 0.000 | 0.002 | 0.003 | 0.359 | 2.785 |
| AST | -0.002 | 0.000 | -0.067 | -7.918 | 0.000 | -0.002 | -0.001 | 0.402 | 2.486 |
| GGT | 0.002 | 0.000 | 0.124 | 20.185 | 0.000 | 0.001 | 0.002 | 0.768 | 1.303 |

Dependent Variable: MASLD.

Abbreviations: ALT, Alanine Aminotransferase; AST, Aspartate Aminotransferase; BMI, Body mass index; GGT,Gamma-Glutamyl Transferase; HDL, High density ;PIR, Poverty income ratio; Sig, significance; TG, triglyceride; lipoprotein; VIF, variance inflation factor ;WC, waist circumference;

| **TABLE S2(A)** The Variables in the Equation for SII and MASLD in Model 1 | | | | | | | | | |
| --- | --- | --- | --- | --- | --- | --- | --- | --- | --- |
|  | | B | S.E. | Wald | df | Sig. | OR | 95% C.I.for OR | |
|  |  |  |  |  |  |  |  | Lower | Upper |
| Step 1^a^ | SII |  |  | 102.778 | 3 | 0.000 |  |  |  |
|  | SII (1) | 0.246 | 0.048 | 26.404 | 1 | 0.000 | 1.278 | 1.164 | 1.404 |
|  | SII (2) | 0.263 | 0.048 | 30.212 | 1 | 0.000 | 1.300 | 1.184 | 1.428 |
|  | SII (3) | 0.483 | 0.048 | 102.606 | 1 | 0.000 | 1.621 | 1.476 | 1.780 |
|  | Constant | -0.441 | 0.034 | 166.956 | 1 | 0.000 | 0.643 |  |  |
| a. Variable(s) entered on step 1: SII . | | | | | | | | | |

| **TABLE S2(B)** The Variables in the Equation for SIRI and MASLD in Model 1 | | | | | | | | | |
| --- | --- | --- | --- | --- | --- | --- | --- | --- | --- |
|  | | B | S.E. | Wald | df | Sig. | OR | 95% C.I.for OR | |
|  |  |  |  |  |  |  |  | Lower | Upper |
| Step 1^a^ | SIRI |  |  | 205.194 | 3 | 0.000 |  |  |  |
|  | SIRI(1) | 0.297 | 0.048 | 37.954 | 1 | 0.000 | 1.346 | 1.224 | 1.479 |
|  | SIRI(2) | 0.498 | 0.048 | 107.700 | 1 | 0.000 | 1.646 | 1.498 | 1.808 |
|  | SIRI (3) | 0.651 | 0.048 | 183.788 | 1 | 0.000 | 1.918 | 1.745 | 2.107 |
|  | Constant | -0.556 | 0.035 | 259.172 | 1 | 0.000 | 0.573 |  |  |
| a. Variable(s) entered on step 1: SIRI. | | | | | | | | | |

| **TABLE S2(C)** The Variables in the Equation for LMR and MASLD in Model 1 | | | | | | | | | |
| --- | --- | --- | --- | --- | --- | --- | --- | --- | --- |
|  | | B | S.E. | Wald | df | Sig. | OR | 95% C.I.for OR | |
|  |  |  |  |  |  |  |  | Lower | Upper |
| Step 1^a^ | LMR |  |  | 0.701 | 3 | 0.873 |  |  |  |
|  | LMR(1) | -0.012 | 0.047 | 0.062 | 1 | 0.804 | 0.988 | 0.901 | 1.084 |
|  | LMR(2) | -0.018 | 0.046 | 0.154 | 1 | 0.695 | 0.982 | 0.898 | 1.074 |
|  | LMR(3) | 0.020 | 0.048 | 0.169 | 1 | 0.681 | 1.020 | 0.928 | 1.121 |
|  | Constant | -0.188 | 0.033 | 33.504 | 1 | 0.000 | 0.828 |  |  |
| a. Variable(s) entered on step 1: LMR . | | | | | | | | | |

| **TABLE S2(D)** The Variables in the Equation for NLR and MASLD in Model 1 | | | | | | | | | |
| --- | --- | --- | --- | --- | --- | --- | --- | --- | --- |
|  | | B | S.E. | Wald | df | Sig. | OR | 95% C.I.for OR | |
|  |  |  |  |  |  |  |  | Lower | Upper |
| Step 1^a^ | NLR |  |  | 70.384 | 3 | 0.000 |  |  |  |
|  | NLR (1) | 0.184 | 0.048 | 14.818 | 1 | 0.000 | 1.202 | 1.094 | 1.319 |
|  | NLR (2) | 0.250 | 0.048 | 27.464 | 1 | 0.000 | 1.284 | 1.169 | 1.409 |
|  | NLR(3) | 0.393 | 0.048 | 68.195 | 1 | 0.000 | 1.482 | 1.350 | 1.626 |
|  | Constant | -0.399 | 0.034 | 138.029 | 1 | 0.000 | 0.671 |  |  |
| a. Variable(s) entered on step 1: NLR . | | | | | | | | | |

| **TABLE S2(E)** The Variables in the Equation for PLR and MASLD in Model 1 | | | | | | | | | |
| --- | --- | --- | --- | --- | --- | --- | --- | --- | --- |
|  | | B | S.E. | Wald | df | Sig. | OR | 95% C.I.for OR | |
|  |  |  |  |  |  |  |  | Lower | Upper |
| Step 1^a^ | PLR |  |  | 84.042 | 3 | 0.000 |  |  |  |
|  | PLR(1) | 0.098 | 0.048 | 4.145 | 1 | 0.042 | 1.103 | 1.004 | 1.211 |
|  | PLR(2) | 0.264 | 0.047 | 30.882 | 1 | 0.000 | 1.302 | 1.186 | 1.428 |
|  | PLR(3) | 0.402 | 0.048 | 71.375 | 1 | 0.000 | 1.495 | 1.361 | 1.641 |
|  | Constant | -0.384 | 0.034 | 128.161 | 1 | 0.000 | 0.681 |  |  |
| a. Variable(s) entered on step 1: PLR . | | | | | | | | | |

| **TABLE S2(F)** The Variables in the Equation for NPAR and MASLD in Model 1 | | | | | | | | | |
| --- | --- | --- | --- | --- | --- | --- | --- | --- | --- |
|  | | B | S.E. | Wald | df | Sig. | OR | 95% C.I.for OR | |
|  |  |  |  |  |  |  |  | Lower | Upper |
| Step 1^a^ | NPAR |  |  | 236.986 | 3 | 0.000 |  |  |  |
|  | NPAR (1) | 0.266 | 0.048 | 30.272 | 1 | 0.000 | 1.304 | 1.187 | 1.434 |
|  | NPAR(2) | 0.460 | 0.048 | 91.575 | 1 | 0.000 | 1.584 | 1.442 | 1.741 |
|  | NPAR(3) | 0.714 | 0.048 | 220.084 | 1 | 0.000 | 2.043 | 1.859 | 2.245 |
|  | Constant | -0.555 | 0.035 | 256.686 | 1 | 0.000 | 0.574 |  |  |
| a. Variable(s) entered on step 1: NPAR. | | | | | | | | | |

| **TABLE S3(A)** The Variables in the Equation for SII and MASLD in Model 2 | | | | | | | | | |
| --- | --- | --- | --- | --- | --- | --- | --- | --- | --- |
|  | | B | S.E. | Wald | df | Sig. | OR | 95% C.I.for OR | |
|  |  |  |  |  |  |  |  | Lower | Upper |
| Step 1^a^ | Age | 0.010 | 0.001 | 72.794 | 1 | 0.000 | 1.010 | 1.008 | 1.012 |
|  | Gender(1) | -0.382 | 0.035 | 118.443 | 1 | 0.000 | 0.683 | 0.637 | 0.731 |
|  | Race/Ethnicity |  |  | 273.118 | 4 | 0.000 |  |  |  |
|  | Race/Ethnicity(1) | -0.389 | 0.067 | 33.351 | 1 | 0.000 | 0.678 | 0.594 | 0.773 |
|  | Race/Ethnicity(2) | -0.533 | 0.054 | 96.056 | 1 | 0.000 | 0.587 | 0.527 | 0.653 |
|  | Race/Ethnicity(3) | -0.425 | 0.061 | 49.074 | 1 | 0.000 | 0.654 | 0.581 | 0.737 |
|  | Race/Ethnicity(4) | -1.161 | 0.071 | 267.184 | 1 | 0.000 | 0.313 | 0.273 | 0.360 |
|  | PIR(1) | 0.167 | 0.040 | 17.737 | 1 | 0.000 | 1.181 | 1.093 | 1.277 |
|  | Education(1) | -0.034 | 0.044 | 0.602 | 1 | 0.438 | 0.967 | 0.887 | 1.053 |
|  | Marital status |  |  | 13.611 | 2 | 0.001 |  |  |  |
|  | Marital status(1) | 0.175 | 0.062 | 8.062 | 1 | 0.005 | 1.192 | 1.056 | 1.345 |
|  | Marital status(2) | 0.186 | 0.051 | 13.455 | 1 | 0.000 | 1.204 | 1.090 | 1.330 |
|  | Health insurance(1) | -0.084 | 0.046 | 3.364 | 1 | 0.067 | 0.920 | 0.841 | 1.006 |
|  | SII |  |  | 96.370 | 3 | 0.000 |  |  |  |
|  | SII (1) | 0.244 | 0.049 | 24.591 | 1 | 0.000 | 1.276 | 1.159 | 1.406 |
|  | SII (2) | 0.269 | 0.049 | 29.730 | 1 | 0.000 | 1.309 | 1.188 | 1.442 |
|  | SII (3) | 0.487 | 0.050 | 96.154 | 1 | 0.000 | 1.628 | 1.477 | 1.794 |
|  | Constant | -0.417 | 0.092 | 20.315 | 1 | 0.000 | 0.659 |  |  |
| a. Variable(s) entered on step 1: gender, age, race/ethnicity, PIR, education, marital status, health insurance,and SII. | | | | | | | | | |

| **TABLE S3(B)** The Variables in the Equation for SIRI and MASLD in Model 2 | | | | | | | | | |
| --- | --- | --- | --- | --- | --- | --- | --- | --- | --- |
|  | | B | S.E. | Wald | df | Sig. | OR | 95% C.I.for OR | |
|  |  |  |  |  |  |  |  | Lower | Upper |
| Step 1^a^ | Age | 0.009 | 0.001 | 57.050 | 1 | 0.000 | 1.009 | 1.007 | 1.011 |
|  | Gender(1) | -0.306 | 0.035 | 75.446 | 1 | 0.000 | 0.737 | 0.687 | 0.789 |
|  | Race/Ethnicity |  |  | 272.345 | 4 | 0.000 |  |  |  |
|  | Race/Ethnicity(1) | -0.390 | 0.067 | 33.415 | 1 | 0.000 | 0.677 | 0.594 | 0.773 |
|  | Race/Ethnicity(2) | -0.561 | 0.055 | 105.358 | 1 | 0.000 | 0.571 | 0.513 | 0.635 |
|  | Race/Ethnicity(3) | -0.410 | 0.061 | 45.606 | 1 | 0.000 | 0.664 | 0.589 | 0.747 |
|  | Race/Ethnicity(4) | -1.150 | 0.071 | 261.639 | 1 | 0.000 | 0.317 | 0.275 | 0.364 |
|  | PIR(1) | 0.155 | 0.040 | 15.242 | 1 | 0.000 | 1.167 | 1.080 | 1.262 |
|  | Education(1) | -0.033 | 0.044 | 0.556 | 1 | 0.456 | 0.968 | 0.888 | 1.055 |
|  | Marital status |  |  | 16.129 | 2 | 0.000 |  |  |  |
|  | Marital status(1) | 0.188 | 0.062 | 9.227 | 1 | 0.002 | 1.206 | 1.069 | 1.362 |
|  | Marital status(2) | 0.203 | 0.051 | 16.007 | 1 | 0.000 | 1.225 | 1.109 | 1.353 |
|  | Health insurance(1) | -0.081 | 0.046 | 3.110 | 1 | 0.078 | 0.923 | 0.844 | 1.009 |
|  | SIRI |  |  | 124.294 | 3 | 0.000 |  |  |  |
|  | SIRI(1) | 0.252 | 0.050 | 25.808 | 1 | 0.000 | 1.287 | 1.167 | 1.418 |
|  | SIRI(2) | 0.425 | 0.050 | 72.445 | 1 | 0.000 | 1.530 | 1.387 | 1.688 |
|  | SIRI (3) | 0.534 | 0.051 | 109.930 | 1 | 0.000 | 1.706 | 1.544 | 1.885 |
|  | Constant | -0.459 | 0.093 | 24.539 | 1 | 0.000 | 0.632 |  |  |
| a. Variable(s) entered on step 1: gender, age, race/ethnicity, PIR, education, marital status, health insurance,and SIRI. | | | | | | | | | |

| **TABLE S3(C)** The Variables in the Equation for LMR and MASLD in Model 2 | | | | | | | | | |
| --- | --- | --- | --- | --- | --- | --- | --- | --- | --- |
|  | | B | S.E. | Wald | df | Sig. | OR | 95% C.I.for OR | |
|  |  |  |  |  |  |  |  | Lower | Upper |
| Step 1^a^ | Age | 0.011 | 0.001 | 92.972 | 1 | 0.000 | 1.011 | 1.009 | 1.014 |
|  | Gender(1) | -0.394 | 0.036 | 121.878 | 1 | 0.000 | 0.675 | 0.629 | 0.723 |
|  | Race/Ethnicity |  |  | 286.308 | 4 | 0.000 |  |  |  |
|  | Race/Ethnicity(1) | -0.378 | 0.067 | 31.563 | 1 | 0.000 | 0.685 | 0.601 | 0.782 |
|  | Race/Ethnicity(2) | -0.476 | 0.054 | 76.453 | 1 | 0.000 | 0.621 | 0.558 | 0.691 |
|  | Race/Ethnicity(3) | -0.486 | 0.060 | 64.885 | 1 | 0.000 | 0.615 | 0.547 | 0.692 |
|  | Race/Ethnicity(4) | -1.195 | 0.071 | 283.433 | 1 | 0.000 | 0.303 | 0.263 | 0.348 |
|  | PIR(1) | 0.169 | 0.039 | 18.372 | 1 | 0.000 | 1.184 | 1.096 | 1.280 |
|  | Education(1) | -0.023 | 0.044 | 0.277 | 1 | 0.599 | 0.977 | 0.897 | 1.065 |
|  | Marital status |  |  | 12.040 | 2 | 0.002 |  |  |  |
|  | Marital status(1) | 0.182 | 0.062 | 8.712 | 1 | 0.003 | 1.199 | 1.063 | 1.353 |
|  | Marital status(2) | 0.171 | 0.051 | 11.332 | 1 | 0.001 | 1.186 | 1.074 | 1.310 |
|  | Health insurance(1) | -0.084 | 0.046 | 3.416 | 1 | 0.065 | 0.919 | 0.841 | 1.005 |
|  | LMR |  |  | 29.321 | 3 | 0.000 |  |  |  |
|  | LMR(1) | 0.105 | 0.049 | 4.539 | 1 | 0.033 | 1.110 | 1.008 | 1.222 |
|  | LMR(2) | 0.163 | 0.048 | 11.439 | 1 | 0.001 | 1.178 | 1.071 | 1.295 |
|  | LMR(3) | 0.280 | 0.053 | 28.209 | 1 | 0.000 | 1.323 | 1.193 | 1.467 |
|  | Constant | -0.376 | 0.096 | 15.422 | 1 | 0.000 | 0.687 |  |  |
| a. Variable(s) entered on step 1: gender, age, race/ethnicity, PIR, education, marital status, health insurance,and LMR. | | | | | | | | | |

| **TABLE S3(D)** The Variables in the Equation for NLR and MASLD in Model 2 | | | | | | | | | |
| --- | --- | --- | --- | --- | --- | --- | --- | --- | --- |
|  | | B | S.E. | Wald | df | Sig. | OR | 95% C.I.for OR | |
|  |  |  |  |  |  |  |  | Lower | Upper |
| Step 1^a^ | Age | 0.009 | 0.001 | 65.922 | 1 | 0.000 | 1.009 | 1.007 | 1.012 |
|  | Gender(1) | -0.349 | 0.035 | 100.192 | 1 | 0.000 | 0.705 | 0.659 | 0.755 |
|  | Race/Ethnicity |  |  | 276.068 | 4 | 0.000 |  |  |  |
|  | Race/Ethnicity(1) | -0.384 | 0.067 | 32.694 | 1 | 0.000 | 0.681 | 0.597 | 0.777 |
|  | Race/Ethnicity(2) | -0.525 | 0.054 | 93.465 | 1 | 0.000 | 0.592 | 0.532 | 0.658 |
|  | Race/Ethnicity(3) | -0.433 | 0.061 | 50.882 | 1 | 0.000 | 0.649 | 0.576 | 0.731 |
|  | Race/Ethnicity(4) | -1.167 | 0.071 | 271.125 | 1 | 0.000 | 0.311 | 0.271 | 0.358 |
|  | PIR(1) | 0.167 | 0.040 | 17.970 | 1 | 0.000 | 1.182 | 1.094 | 1.277 |
|  | Education(1) | -0.034 | 0.044 | 0.624 | 1 | 0.430 | 0.966 | 0.887 | 1.052 |
|  | Marital status |  |  | 13.878 | 2 | 0.001 |  |  |  |
|  | Marital status(1) | 0.185 | 0.062 | 9.043 | 1 | 0.003 | 1.204 | 1.067 | 1.358 |
|  | Marital status(2) | 0.186 | 0.051 | 13.489 | 1 | 0.000 | 1.204 | 1.091 | 1.330 |
|  | Health insurance(1) | -0.079 | 0.046 | 3.023 | 1 | 0.082 | 0.924 | 0.845 | 1.010 |
|  | NLR |  |  | 37.908 | 3 | 0.000 |  |  |  |
|  | NLR (1) | 0.164 | 0.049 | 11.049 | 1 | 0.001 | 1.178 | 1.069 | 1.297 |
|  | NLR (2) | 0.204 | 0.049 | 17.011 | 1 | 0.000 | 1.226 | 1.113 | 1.350 |
|  | NLR(3) | 0.303 | 0.050 | 36.650 | 1 | 0.000 | 1.354 | 1.227 | 1.494 |
|  | Constant | -0.333 | 0.092 | 13.149 | 1 | 0.000 | 0.717 |  |  |
| a. Variable(s) entered on step 1: gender, age, race/ethnicity, PIR, education, marital status, health insurance,and NLR. | | | | | | | | | |

| **TABLE S3(E)** The Variables in the Equation for PLR and MASLD in Model 2 | | | | | | | | | |
| --- | --- | --- | --- | --- | --- | --- | --- | --- | --- |
|  | | B | S.E. | Wald | df | Sig. | OR | 95% C.I.for OR | |
|  |  |  |  |  |  |  |  | Lower | Upper |
| Step 1^a^ | Age | 0.011 | 0.001 | 86.371 | 1 | 0.000 | 1.011 | 1.008 | 1.013 |
|  | Gender(1) | -0.319 | 0.035 | 82.665 | 1 | 0.000 | 0.727 | 0.678 | 0.778 |
|  | Race/Ethnicity |  |  | 283.533 | 4 | 0.000 |  |  |  |
|  | Race/Ethnicity(1) | -0.377 | 0.067 | 31.341 | 1 | 0.000 | 0.686 | 0.601 | 0.783 |
|  | Race/Ethnicity(2) | -0.489 | 0.054 | 81.248 | 1 | 0.000 | 0.613 | 0.551 | 0.682 |
|  | Race/Ethnicity(3) | -0.476 | 0.060 | 62.181 | 1 | 0.000 | 0.621 | 0.552 | 0.699 |
|  | Race/Ethnicity(4) | -1.190 | 0.071 | 280.837 | 1 | 0.000 | 0.304 | 0.265 | 0.350 |
|  | PIR(1) | 0.158 | 0.040 | 15.974 | 1 | 0.000 | 1.171 | 1.084 | 1.266 |
|  | Education(1) | -0.017 | 0.044 | 0.157 | 1 | 0.692 | 0.983 | 0.902 | 1.071 |
|  | Marital status |  |  | 13.283 | 2 | 0.001 |  |  |  |
|  | Marital status(1) | 0.189 | 0.062 | 9.343 | 1 | 0.002 | 1.207 | 1.070 | 1.363 |
|  | Marital status(2) | 0.180 | 0.051 | 12.634 | 1 | 0.000 | 1.197 | 1.084 | 1.322 |
|  | Health insurance(1) | -0.082 | 0.046 | 3.225 | 1 | 0.073 | 0.921 | 0.843 | 1.008 |
|  | PLR |  |  | 75.771 | 3 | 0.000 |  |  |  |
|  | PLR(1) | 0.117 | 0.049 | 5.688 | 1 | 0.017 | 1.124 | 1.021 | 1.238 |
|  | PLR(2) | 0.281 | 0.049 | 33.278 | 1 | 0.000 | 1.325 | 1.204 | 1.458 |
|  | PLR(3) | 0.396 | 0.049 | 64.647 | 1 | 0.000 | 1.485 | 1.349 | 1.636 |
|  | Constant | -0.457 | 0.095 | 23.111 | 1 | 0.000 | 0.633 |  |  |
| a. Variable(s) entered on step 1: gender, age, race/ethnicity, PIR, education, marital status, health insurance,and PLR. | | | | | | | | | |

| **TABLE S3(F)** The Variables in the Equation for NPAR and MASLD in Model 2 | | | | | | | | | |
| --- | --- | --- | --- | --- | --- | --- | --- | --- | --- |
|  | | B | S.E. | Wald | df | Sig. | OR | 95% C.I.for OR | |
|  |  |  |  |  |  |  |  | Lower | Upper |
| Step 1^a^ | Age | 0.008 | 0.001 | 49.721 | 1 | 0.000 | 1.008 | 1.006 | 1.011 |
|  | Gender(1) | -0.418 | 0.035 | 139.310 | 1 | 0.000 | 0.658 | 0.614 | 0.706 |
|  | Race/Ethnicity |  |  | 264.534 | 4 | 0.000 |  |  |  |
|  | Race/Ethnicity(1) | -0.380 | 0.068 | 31.618 | 1 | 0.000 | 0.684 | 0.599 | 0.781 |
|  | Race/Ethnicity(2) | -0.524 | 0.055 | 92.516 | 1 | 0.000 | 0.592 | 0.532 | 0.659 |
|  | Race/Ethnicity(3) | -0.427 | 0.061 | 49.295 | 1 | 0.000 | 0.653 | 0.579 | 0.735 |
|  | Race/Ethnicity(4) | -1.148 | 0.071 | 259.260 | 1 | 0.000 | 0.317 | 0.276 | 0.365 |
|  | PIR(1) | 0.156 | 0.040 | 15.392 | 1 | 0.000 | 1.169 | 1.081 | 1.263 |
|  | Education(1) | -0.033 | 0.044 | 0.578 | 1 | 0.447 | 0.967 | 0.887 | 1.054 |
|  | Marital status |  |  | 13.010 | 2 | 0.001 |  |  |  |
|  | Marital status(1) | 0.174 | 0.062 | 7.849 | 1 | 0.005 | 1.190 | 1.054 | 1.343 |
|  | Marital status(2) | 0.182 | 0.051 | 12.827 | 1 | 0.000 | 1.200 | 1.086 | 1.326 |
|  | Health insurance(1) | -0.072 | 0.046 | 2.495 | 1 | 0.114 | 0.930 | 0.850 | 1.018 |
|  | NPAR |  |  | 203.259 | 3 | 0.000 |  |  |  |
|  | NPAR (1) | 0.234 | 0.050 | 22.358 | 1 | 0.000 | 1.264 | 1.147 | 1.393 |
|  | NPAR(2) | 0.418 | 0.050 | 70.692 | 1 | 0.000 | 1.519 | 1.378 | 1.674 |
|  | NPAR(3) | 0.692 | 0.050 | 189.640 | 1 | 0.000 | 1.998 | 1.811 | 2.205 |
|  | Constant | -0.407 | 0.092 | 19.745 | 1 | 0.000 | 0.666 |  |  |
| a. Variable(s) entered on step 1: gender, age, race/ethnicity, PIR, education, marital status, health insurance,and NPAR. | | | | | | | | | |

| **TABLE S4(A**) The Variables in the Equation for SII and MASLD in Model 3 | | | | | | | | | |
| --- | --- | --- | --- | --- | --- | --- | --- | --- | --- |
|  | | B | S.E. | Wald | df | Sig. | OR | 95% C.I.for OR | |
|  |  |  |  |  |  |  |  | Lower | Upper |
| Step 1^a^ | Age | 0.002 | 0.002 | 0.753 | 1 | 0.386 | 1.002 | 0.997 | 1.007 |
|  | Gender(1) | -1.351 | 0.082 | 274.011 | 1 | 0.000 | 0.259 | 0.221 | 0.304 |
|  | Race/Ethnicity |  |  | 63.003 | 4 | 0.000 |  |  |  |
|  | Race/Ethnicity(1) | -0.369 | 0.114 | 10.562 | 1 | 0.001 | 0.691 | 0.553 | 0.864 |
|  | Race/Ethnicity(2) | -0.338 | 0.095 | 12.601 | 1 | 0.000 | 0.714 | 0.592 | 0.860 |
|  | Race/Ethnicity(3) | -0.808 | 0.105 | 59.400 | 1 | 0.000 | 0.446 | 0.363 | 0.548 |
|  | Race/Ethnicity(4) | -0.408 | 0.123 | 11.040 | 1 | 0.001 | 0.665 | 0.522 | 0.846 |
|  | PIR(1) | 0.041 | 0.068 | 0.361 | 1 | 0.548 | 1.042 | 0.912 | 1.190 |
|  | Education(1) | 0.029 | 0.075 | 0.145 | 1 | 0.703 | 1.029 | 0.888 | 1.192 |
|  | Marital status |  |  | 2.197 | 2 | 0.333 |  |  |  |
|  | Marital status(1) | -0.121 | 0.105 | 1.331 | 1 | 0.249 | 0.886 | 0.721 | 1.088 |
|  | Marital status(2) | -0.131 | 0.089 | 2.174 | 1 | 0.140 | 0.877 | 0.737 | 1.044 |
|  | Health insurance(1) | 0.078 | 0.078 | 1.002 | 1 | 0.317 | 1.081 | 0.928 | 1.260 |
|  | Alcohol use(1) | -0.006 | 0.062 | 0.008 | 1 | 0.929 | 0.994 | 0.881 | 1.122 |
|  | Tobacco use(1) | -0.107 | 0.064 | 2.855 | 1 | 0.091 | 0.898 | 0.793 | 1.017 |
|  | Hypertension(1) | 0.217 | 0.068 | 10.207 | 1 | 0.001 | 1.242 | 1.088 | 1.419 |
|  | Diabetes(1) | 0.858 | 0.081 | 111.977 | 1 | 0.000 | 2.359 | 2.012 | 2.766 |
|  | Cardiovascular diseases(1) | 0.116 | 0.069 | 2.834 | 1 | 0.092 | 1.123 | 0.981 | 1.286 |
|  | WC01(1) | 1.756 | 0.092 | 363.881 | 1 | 0.000 | 5.788 | 4.833 | 6.932 |
|  | PA01(1) | -0.224 | 0.062 | 12.930 | 1 | 0.000 | 0.799 | 0.707 | 0.903 |
|  | BMI |  |  | 2102.091 | 2 | 0.000 |  |  |  |
|  | BMI(1) | 2.331 | 0.124 | 354.504 | 1 | 0.000 | 10.289 | 8.072 | 13.115 |
|  | BMI(2) | 5.437 | 0.143 | 1446.357 | 1 | 0.000 | 229.707 | 173.576 | 303.991 |
|  | TG(1) | 2.278 | 0.081 | 791.453 | 1 | 0.000 | 9.755 | 8.324 | 11.433 |
|  | HDL(1) | -1.123 | 0.229 | 24.023 | 1 | 0.000 | 0.325 | 0.208 | 0.510 |
|  | ALT | 0.034 | 0.003 | 101.556 | 1 | 0.000 | 1.034 | 1.028 | 1.041 |
|  | AST | -0.020 | 0.003 | 38.998 | 1 | 0.000 | 0.980 | 0.974 | 0.986 |
|  | GGT | 0.039 | 0.002 | 494.338 | 1 | 0.000 | 1.040 | 1.036 | 1.043 |
|  | SII |  |  | 20.650 | 3 | 0.000 |  |  |  |
|  | SII (1) | 0.247 | 0.085 | 8.467 | 1 | 0.004 | 1.281 | 1.084 | 1.513 |
|  | SII (2) | 0.222 | 0.085 | 6.818 | 1 | 0.009 | 1.249 | 1.057 | 1.476 |
|  | SII (3) | 0.386 | 0.086 | 20.246 | 1 | 0.000 | 1.471 | 1.244 | 1.741 |
|  | Constant | -5.548 | 0.244 | 517.913 | 1 | 0.000 | 0.004 |  |  |
| a. Variable(s) entered on step 1: gender, age, race/ethnicity, PIR, education, marital status, health insurance, tobacco use, alcohol use, hypertension, T2DM, cardiovascular disease, WC, PA, body mass index (BMI), TG, HDL, ALT, AST, GGT,and SII. | | | | | | | | | |
|  | | | | | | | | | |

| **TABLE S4(B)** The Variables in the Equation for SIRI and MASLD in Model 3 | | | | | | | | | |
| --- | --- | --- | --- | --- | --- | --- | --- | --- | --- |
|  | | B | S.E. | Wald | df | Sig. | OR | 95% C.I.for OR | |
|  |  |  |  |  |  |  |  | Lower | Upper |
| Step 1^a^ | Age | 0.001 | 0.002 | 0.269 | 1 | 0.604 | 1.001 | 0.997 | 1.006 |
|  | Gender(1) | -1.307 | 0.082 | 254.392 | 1 | 0.000 | 0.271 | 0.231 | 0.318 |
|  | Race/Ethnicity |  |  | 64.635 | 4 | 0.000 |  |  |  |
|  | Race/Ethnicity(1) | -0.366 | 0.113 | 10.422 | 1 | 0.001 | 0.693 | 0.555 | 0.866 |
|  | Race/Ethnicity(2) | -0.348 | 0.095 | 13.360 | 1 | 0.000 | 0.706 | 0.586 | 0.851 |
|  | Race/Ethnicity(3) | -0.822 | 0.105 | 61.435 | 1 | 0.000 | 0.440 | 0.358 | 0.540 |
|  | Race/Ethnicity(4) | -0.412 | 0.123 | 11.260 | 1 | 0.001 | 0.662 | 0.521 | 0.843 |
|  | PIR(1) | 0.036 | 0.068 | 0.286 | 1 | 0.593 | 1.037 | 0.908 | 1.184 |
|  | Education(1) | 0.035 | 0.075 | 0.217 | 1 | 0.641 | 1.036 | 0.894 | 1.200 |
|  | Marital status |  |  | 1.873 | 2 | 0.392 |  |  |  |
|  | Marital status(1) | -0.109 | 0.105 | 1.082 | 1 | 0.298 | 0.897 | 0.730 | 1.101 |
|  | Marital status(2) | -0.121 | 0.089 | 1.863 | 1 | 0.172 | 0.886 | 0.745 | 1.054 |
|  | Health insurance(1) | 0.077 | 0.078 | 0.983 | 1 | 0.321 | 1.081 | 0.927 | 1.259 |
|  | Alcohol use(1) | -0.004 | 0.062 | 0.005 | 1 | 0.946 | 0.996 | 0.883 | 1.124 |
|  | Tobacco use(1) | -0.109 | 0.064 | 2.957 | 1 | 0.086 | 0.896 | 0.791 | 1.015 |
|  | Hypertension(1) | 0.214 | 0.068 | 9.954 | 1 | 0.002 | 1.239 | 1.085 | 1.415 |
|  | Diabetes(1) | 0.863 | 0.081 | 113.176 | 1 | 0.000 | 2.370 | 2.022 | 2.778 |
|  | Cardiovascular diseases(1) | 0.108 | 0.069 | 2.429 | 1 | 0.119 | 1.114 | 0.973 | 1.275 |
|  | WC01(1) | 1.763 | 0.092 | 366.386 | 1 | 0.000 | 5.833 | 4.869 | 6.987 |
|  | PA01(1) | -0.228 | 0.062 | 13.402 | 1 | 0.000 | 0.796 | 0.705 | 0.899 |
|  | BMI |  |  | 2100.603 | 2 | 0.000 |  |  |  |
|  | BMI(1) | 2.330 | 0.124 | 354.005 | 1 | 0.000 | 10.282 | 8.066 | 13.107 |
|  | BMI(2) | 5.428 | 0.143 | 1443.153 | 1 | 0.000 | 227.674 | 172.065 | 301.255 |
|  | TG(1) | 2.279 | 0.081 | 792.182 | 1 | 0.000 | 9.766 | 8.333 | 11.446 |
|  | HDL(1) | -1.113 | 0.229 | 23.593 | 1 | 0.000 | 0.329 | 0.210 | 0.515 |
|  | ALT | 0.033 | 0.003 | 96.919 | 1 | 0.000 | 1.034 | 1.027 | 1.041 |
|  | AST | -0.020 | 0.003 | 35.876 | 1 | 0.000 | 0.980 | 0.974 | 0.987 |
|  | GGT | 0.039 | 0.002 | 491.641 | 1 | 0.000 | 1.040 | 1.036 | 1.043 |
|  | SIRI |  |  | 12.841 | 3 | 0.005 |  |  |  |
|  | SIRI(1) | 0.059 | 0.084 | 0.502 | 1 | 0.479 | 1.061 | 0.900 | 1.251 |
|  | SIRI(2) | 0.235 | 0.085 | 7.660 | 1 | 0.006 | 1.265 | 1.071 | 1.495 |
|  | SIRI (3) | 0.256 | 0.087 | 8.619 | 1 | 0.003 | 1.292 | 1.089 | 1.532 |
|  | Constant | -5.437 | 0.242 | 504.740 | 1 | 0.000 | 0.004 |  |  |
| a. Variable(s) entered on step 1: gender, age, race/ethnicity, PIR, education, marital status, health insurance, tobacco use, alcohol use, hypertension, T2DM, cardiovascular disease, WC, PA, body mass index (BMI), TG, HDL, ALT, AST, GGT,and SIRI. | | | | | | | | | |

| **TABLE S4(C)** The Variables in the Equation for LMR and MASLD in Model 3 | | | | | | | | | |
| --- | --- | --- | --- | --- | --- | --- | --- | --- | --- |
|  | | B | S.E. | Wald | df | Sig. | OR | 95% C.I.for OR | |
|  |  |  |  |  |  |  |  | Lower | Upper |
| Step 1^a^ | Age | 0.002 | 0.002 | 0.978 | 1 | 0.323 | 1.002 | 0.998 | 1.007 |
|  | Gender(1) | -1.373 | 0.083 | 275.677 | 1 | 0.000 | 0.253 | 0.215 | 0.298 |
|  | Race/Ethnicity |  |  | 80.507 | 4 | 0.000 |  |  |  |
|  | Race/Ethnicity(1) | -0.372 | 0.114 | 10.763 | 1 | 0.001 | 0.689 | 0.552 | 0.861 |
|  | Race/Ethnicity(2) | -0.299 | 0.095 | 9.828 | 1 | 0.002 | 0.742 | 0.615 | 0.894 |
|  | Race/Ethnicity(3) | -0.877 | 0.104 | 71.032 | 1 | 0.000 | 0.416 | 0.339 | 0.510 |
|  | Race/Ethnicity(4) | -0.438 | 0.123 | 12.719 | 1 | 0.000 | 0.645 | 0.507 | 0.821 |
|  | PIR(1) | 0.041 | 0.068 | 0.365 | 1 | 0.546 | 1.042 | 0.912 | 1.190 |
|  | Education(1) | 0.043 | 0.075 | 0.321 | 1 | 0.571 | 1.043 | 0.901 | 1.209 |
|  | Marital status |  |  | 2.628 | 2 | 0.269 |  |  |  |
|  | Marital status(1) | -0.124 | 0.105 | 1.401 | 1 | 0.237 | 0.883 | 0.719 | 1.085 |
|  | Marital status(2) | -0.144 | 0.089 | 2.626 | 1 | 0.105 | 0.866 | 0.728 | 1.031 |
|  | Health insurance(1) | 0.081 | 0.078 | 1.080 | 1 | 0.299 | 1.084 | 0.931 | 1.264 |
|  | Alcohol use(1) | 0.002 | 0.062 | 0.001 | 1 | 0.971 | 1.002 | 0.888 | 1.131 |
|  | Tobacco use(1) | -0.124 | 0.063 | 3.798 | 1 | 0.051 | 0.884 | 0.781 | 1.001 |
|  | Hypertension(1) | 0.228 | 0.068 | 11.259 | 1 | 0.001 | 1.256 | 1.099 | 1.434 |
|  | Diabetes(1) | 0.874 | 0.081 | 116.229 | 1 | 0.000 | 2.397 | 2.045 | 2.810 |
|  | Cardiovascular diseases(1) | 0.138 | 0.069 | 4.011 | 1 | 0.045 | 1.149 | 1.003 | 1.315 |
|  | WC01(1) | 1.787 | 0.092 | 376.383 | 1 | 0.000 | 5.973 | 4.987 | 7.155 |
|  | PA01(1) | -0.234 | 0.062 | 14.133 | 1 | 0.000 | 0.791 | 0.700 | 0.894 |
|  | BMI |  |  | 2101.813 | 2 | 0.000 |  |  |  |
|  | BMI(1) | 2.320 | 0.124 | 350.598 | 1 | 0.000 | 10.173 | 7.980 | 12.968 |
|  | BMI(2) | 5.417 | 0.143 | 1438.522 | 1 | 0.000 | 225.166 | 170.191 | 297.901 |
|  | TG(1) | 2.261 | 0.081 | 778.530 | 1 | 0.000 | 9.594 | 8.185 | 11.246 |
|  | HDL(1) | -1.090 | 0.230 | 22.429 | 1 | 0.000 | 0.336 | 0.214 | 0.528 |
|  | ALT | 0.033 | 0.003 | 93.890 | 1 | 0.000 | 1.033 | 1.027 | 1.040 |
|  | AST | -0.020 | 0.003 | 33.971 | 1 | 0.000 | 0.980 | 0.974 | 0.987 |
|  | GGT | 0.039 | 0.002 | 493.991 | 1 | 0.000 | 1.040 | 1.036 | 1.043 |
|  | LMR |  |  | 7.782 | 3 | 0.051 |  |  |  |
|  | LMR(1) | 0.131 | 0.084 | 2.421 | 1 | 0.120 | 1.140 | 0.966 | 1.345 |
|  | LMR(2) | 0.200 | 0.083 | 5.726 | 1 | 0.017 | 1.221 | 1.037 | 1.438 |
|  | LMR(3) | 0.225 | 0.090 | 6.219 | 1 | 0.013 | 1.253 | 1.049 | 1.496 |
|  | Constant | -5.455 | 0.244 | 501.743 | 1 | 0.000 | 0.004 |  |  |
| a. Variable(s) entered on step 1: gender, age, race/ethnicity, PIR, education, marital status, health insurance, tobacco use, alcohol use, hypertension, T2DM, cardiovascular disease, WC, PA, body mass index (BMI), TG, HDL, ALT, AST, GGT,and LMR. | | | | | | | | | |

| **TABLE S4(D)** The Variables in the Equation for NLR and MASLD in Model 3 | | | | | | | | | |
| --- | --- | --- | --- | --- | --- | --- | --- | --- | --- |
|  | | B | S.E. | Wald | df | Sig. | OR | 95% C.I.for OR | |
|  |  |  |  |  |  |  |  | Lower | Upper |
| Step 1^a^ | Age | 0.001 | 0.002 | 0.280 | 1 | 0.597 | 1.001 | 0.997 | 1.006 |
|  | Gender(1) | -1.326 | 0.082 | 264.376 | 1 | 0.000 | 0.266 | 0.226 | 0.312 |
|  | Race/Ethnicity |  |  | 64.698 | 4 | 0.000 |  |  |  |
|  | Race/Ethnicity(1) | -0.366 | 0.114 | 10.371 | 1 | 0.001 | 0.694 | 0.555 | 0.867 |
|  | Race/Ethnicity(2) | -0.332 | 0.095 | 12.224 | 1 | 0.000 | 0.717 | 0.595 | 0.864 |
|  | Race/Ethnicity(3) | -0.820 | 0.105 | 60.742 | 1 | 0.000 | 0.440 | 0.358 | 0.541 |
|  | Race/Ethnicity(4) | -0.411 | 0.123 | 11.202 | 1 | 0.001 | 0.663 | 0.521 | 0.844 |
|  | PIR(1) | 0.041 | 0.068 | 0.358 | 1 | 0.550 | 1.041 | 0.912 | 1.189 |
|  | Education(1) | 0.031 | 0.075 | 0.167 | 1 | 0.683 | 1.031 | 0.890 | 1.195 |
|  | Marital status |  |  | 2.233 | 2 | 0.327 |  |  |  |
|  | Marital status(1) | -0.116 | 0.105 | 1.229 | 1 | 0.268 | 0.890 | 0.725 | 1.093 |
|  | Marital status(2) | -0.132 | 0.089 | 2.228 | 1 | 0.136 | 0.876 | 0.736 | 1.042 |
|  | Health insurance(1) | 0.081 | 0.078 | 1.068 | 1 | 0.301 | 1.084 | 0.930 | 1.263 |
|  | Alcohol use(1) | -0.005 | 0.062 | 0.007 | 1 | 0.933 | 0.995 | 0.882 | 1.123 |
|  | Tobacco use(1) | -0.118 | 0.063 | 3.460 | 1 | 0.063 | 0.889 | 0.785 | 1.006 |
|  | Hypertension(1) | 0.216 | 0.068 | 10.134 | 1 | 0.001 | 1.241 | 1.087 | 1.418 |
|  | Diabetes(1) | 0.864 | 0.081 | 113.332 | 1 | 0.000 | 2.373 | 2.024 | 2.782 |
|  | Cardiovascular diseases(1) | 0.116 | 0.069 | 2.837 | 1 | 0.092 | 1.123 | 0.981 | 1.286 |
|  | WC01(1) | 1.767 | 0.092 | 367.912 | 1 | 0.000 | 5.856 | 4.888 | 7.015 |
|  | PA01(1) | -0.226 | 0.062 | 13.177 | 1 | 0.000 | 0.798 | 0.706 | 0.901 |
|  | BMI |  |  | 2104.048 | 2 | 0.000 |  |  |  |
|  | BMI(1) | 2.326 | 0.124 | 352.961 | 1 | 0.000 | 10.237 | 8.032 | 13.049 |
|  | BMI(2) | 5.427 | 0.143 | 1444.389 | 1 | 0.000 | 227.418 | 171.902 | 300.863 |
|  | TG(1) | 2.280 | 0.081 | 792.440 | 1 | 0.000 | 9.775 | 8.340 | 11.456 |
|  | HDL(1) | -1.122 | 0.229 | 23.982 | 1 | 0.000 | 0.326 | 0.208 | 0.510 |
|  | ALT | 0.033 | 0.003 | 97.540 | 1 | 0.000 | 1.034 | 1.027 | 1.041 |
|  | AST | -0.020 | 0.003 | 36.238 | 1 | 0.000 | 0.980 | 0.974 | 0.986 |
|  | GGT | 0.039 | 0.002 | 494.508 | 1 | 0.000 | 1.040 | 1.036 | 1.044 |
|  | NLR |  |  | 9.283 | 3 | 0.026 |  |  |  |
|  | NLR (1) | 0.178 | 0.084 | 4.483 | 1 | 0.034 | 1.195 | 1.013 | 1.408 |
|  | NLR (2) | 0.112 | 0.084 | 1.776 | 1 | 0.183 | 1.119 | 0.948 | 1.320 |
|  | NLR(3) | 0.252 | 0.087 | 8.491 | 1 | 0.004 | 1.287 | 1.086 | 1.525 |
|  | Constant | -5.425 | 0.242 | 503.973 | 1 | 0.000 | 0.004 |  |  |
| a. Variable(s) entered on step 1: gender, age, race/ethnicity, PIR, education, marital status, health insurance, tobacco use, alcohol use, hypertension, T2DM, cardiovascular disease, WC, PA, body mass index (BMI), TG, HDL, ALT, AST, GGT,and NLR. | | | | | | | | | |

| **TABLE S4(E)** The Variables in the Equation for PLR and MASLD in Model 3 | | | | | | | | | |
| --- | --- | --- | --- | --- | --- | --- | --- | --- | --- |
|  | | B | S.E. | Wald | df | Sig. | OR | 95% C.I.for OR | |
|  |  |  |  |  |  |  |  | Lower | Upper |
| Step 1^a^ | Age | 0.002 | 0.002 | 0.404 | 1 | 0.525 | 1.002 | 0.997 | 1.006 |
|  | Gender(1) | -1.328 | 0.082 | 264.743 | 1 | 0.000 | 0.265 | 0.226 | 0.311 |
|  | Race/Ethnicity |  |  | 77.502 | 4 | 0.000 |  |  |  |
|  | Race/Ethnicity(1) | -0.370 | 0.113 | 10.658 | 1 | 0.001 | 0.690 | 0.553 | 0.862 |
|  | Race/Ethnicity(2) | -0.317 | 0.095 | 11.152 | 1 | 0.001 | 0.728 | 0.604 | 0.877 |
|  | Race/Ethnicity(3) | -0.867 | 0.104 | 69.514 | 1 | 0.000 | 0.420 | 0.343 | 0.515 |
|  | Race/Ethnicity(4) | -0.431 | 0.123 | 12.309 | 1 | 0.000 | 0.650 | 0.511 | 0.827 |
|  | PIR(1) | 0.040 | 0.068 | 0.347 | 1 | 0.556 | 1.041 | 0.911 | 1.188 |
|  | Education(1) | 0.042 | 0.075 | 0.308 | 1 | 0.579 | 1.043 | 0.900 | 1.208 |
|  | Marital status |  |  | 2.280 | 2 | 0.320 |  |  |  |
|  | Marital status(1) | -0.113 | 0.105 | 1.161 | 1 | 0.281 | 0.893 | 0.727 | 1.097 |
|  | Marital status(2) | -0.134 | 0.089 | 2.280 | 1 | 0.131 | 0.875 | 0.735 | 1.041 |
|  | Health insurance(1) | 0.083 | 0.078 | 1.141 | 1 | 0.285 | 1.087 | 0.933 | 1.266 |
|  | Alcohol use(1) | -0.001 | 0.062 | 0.000 | 1 | 0.986 | 0.999 | 0.885 | 1.127 |
|  | Tobacco use(1) | -0.120 | 0.063 | 3.594 | 1 | 0.058 | 0.887 | 0.783 | 1.004 |
|  | Hypertension(1) | 0.225 | 0.068 | 11.020 | 1 | 0.001 | 1.253 | 1.097 | 1.431 |
|  | Diabetes(1) | 0.871 | 0.081 | 115.230 | 1 | 0.000 | 2.388 | 2.037 | 2.800 |
|  | Cardiovascular diseases(1) | 0.125 | 0.069 | 3.298 | 1 | 0.069 | 1.133 | 0.990 | 1.297 |
|  | WC01(1) | 1.781 | 0.092 | 374.077 | 1 | 0.000 | 5.935 | 4.955 | 7.109 |
|  | PA01(1) | -0.236 | 0.062 | 14.332 | 1 | 0.000 | 0.790 | 0.699 | 0.893 |
|  | BMI |  |  | 2100.917 | 2 | 0.000 |  |  |  |
|  | BMI(1) | 2.322 | 0.124 | 351.131 | 1 | 0.000 | 10.197 | 7.998 | 13.001 |
|  | BMI(2) | 5.418 | 0.143 | 1438.776 | 1 | 0.000 | 225.433 | 170.386 | 298.264 |
|  | TG(1) | 2.267 | 0.081 | 783.398 | 1 | 0.000 | 9.646 | 8.231 | 11.306 |
|  | HDL(1) | -1.092 | 0.230 | 22.546 | 1 | 0.000 | 0.336 | 0.214 | 0.527 |
|  | ALT | 0.033 | 0.003 | 91.094 | 1 | 0.000 | 1.033 | 1.026 | 1.040 |
|  | AST | -0.020 | 0.004 | 32.144 | 1 | 0.000 | 0.980 | 0.973 | 0.987 |
|  | GGT | 0.039 | 0.002 | 491.436 | 1 | 0.000 | 1.040 | 1.036 | 1.043 |
|  | PLR |  |  | 3.616 | 3 | 0.306 |  |  |  |
|  | PLR(1) | 0.027 | 0.083 | 0.106 | 1 | 0.744 | 1.027 | 0.873 | 1.209 |
|  | PLR(2) | 0.127 | 0.083 | 2.373 | 1 | 0.123 | 1.136 | 0.966 | 1.336 |
|  | PLR(3) | 0.121 | 0.084 | 2.072 | 1 | 0.150 | 1.129 | 0.957 | 1.331 |
|  | Constant | -5.356 | 0.241 | 495.521 | 1 | 0.000 | 0.005 |  |  |
| a. Variable(s) entered on step 1: gender, age, race/ethnicity, PIR, education, marital status, health insurance, tobacco use, alcohol use, hypertension, T2DM, cardiovascular disease, WC, PA, body mass index (BMI), TG, HDL, ALT, AST, GGT,and PLR. | | | | | | | | | |

| **TABLE S4(F)** The Variables in the Equation for NPAR and MASLD in Model 3 | | | | | | | | | |
| --- | --- | --- | --- | --- | --- | --- | --- | --- | --- |
|  | | B | S.E. | Wald | df | Sig. | OR | 95% C.I.for OR | |
|  |  |  |  |  |  |  |  | Lower | Upper |
| Step 1^a^ | Age | 0.001 | 0.002 | 0.294 | 1 | 0.588 | 1.001 | 0.997 | 1.006 |
|  | Gender(1) | -1.347 | 0.082 | 272.516 | 1 | 0.000 | 0.260 | 0.222 | 0.305 |
|  | Race/Ethnicity |  |  | 68.700 | 4 | 0.000 |  |  |  |
|  | Race/Ethnicity(1) | -0.365 | 0.113 | 10.338 | 1 | 0.001 | 0.694 | 0.556 | 0.867 |
|  | Race/Ethnicity(2) | -0.325 | 0.095 | 11.704 | 1 | 0.001 | 0.722 | 0.600 | 0.870 |
|  | Race/Ethnicity(3) | -0.832 | 0.105 | 63.199 | 1 | 0.000 | 0.435 | 0.354 | 0.534 |
|  | Race/Ethnicity(4) | -0.411 | 0.123 | 11.215 | 1 | 0.001 | 0.663 | 0.521 | 0.843 |
|  | PIR(1) | 0.038 | 0.068 | 0.312 | 1 | 0.576 | 1.039 | 0.909 | 1.186 |
|  | Education(1) | 0.033 | 0.075 | 0.197 | 1 | 0.657 | 1.034 | 0.892 | 1.198 |
|  | Marital status |  |  | 2.201 | 2 | 0.333 |  |  |  |
|  | Marital status(1) | -0.116 | 0.105 | 1.216 | 1 | 0.270 | 0.891 | 0.725 | 1.094 |
|  | Marital status(2) | -0.131 | 0.089 | 2.196 | 1 | 0.138 | 0.877 | 0.737 | 1.043 |
|  | Health insurance(1) | 0.083 | 0.078 | 1.121 | 1 | 0.290 | 1.086 | 0.932 | 1.266 |
|  | Alcohol use(1) | -0.004 | 0.062 | 0.004 | 1 | 0.947 | 0.996 | 0.883 | 1.124 |
|  | Tobacco use(1) | -0.116 | 0.064 | 3.314 | 1 | 0.069 | 0.891 | 0.787 | 1.009 |
|  | Hypertension(1) | 0.222 | 0.068 | 10.742 | 1 | 0.001 | 1.249 | 1.093 | 1.427 |
|  | Diabetes(1) | 0.857 | 0.081 | 111.423 | 1 | 0.000 | 2.356 | 2.010 | 2.763 |
|  | Cardiovascular diseases(1) | 0.113 | 0.069 | 2.670 | 1 | 0.102 | 1.119 | 0.978 | 1.282 |
|  | WC01(1) | 1.761 | 0.092 | 365.272 | 1 | 0.000 | 5.816 | 4.855 | 6.967 |
|  | PA01(1) | -0.227 | 0.062 | 13.214 | 1 | 0.000 | 0.797 | 0.706 | 0.901 |
|  | BMI |  |  | 2088.893 | 2 | 0.000 |  |  |  |
|  | BMI(1) | 2.330 | 0.124 | 353.179 | 1 | 0.000 | 10.282 | 8.064 | 13.111 |
|  | BMI(2) | 5.416 | 0.143 | 1436.016 | 1 | 0.000 | 225.031 | 170.052 | 297.785 |
|  | TG(1) | 2.279 | 0.081 | 791.971 | 1 | 0.000 | 9.766 | 8.333 | 11.446 |
|  | HDL(1) | -1.116 | 0.229 | 23.660 | 1 | 0.000 | 0.328 | 0.209 | 0.514 |
|  | ALT | 0.034 | 0.003 | 100.268 | 1 | 0.000 | 1.034 | 1.028 | 1.041 |
|  | AST | -0.020 | 0.003 | 36.967 | 1 | 0.000 | 0.980 | 0.974 | 0.986 |
|  | GGT | 0.039 | 0.002 | 491.210 | 1 | 0.000 | 1.040 | 1.036 | 1.043 |
|  | NPAR |  |  | 9.010 | 3 | 0.029 |  |  |  |
|  | NPAR (1) | 0.097 | 0.085 | 1.295 | 1 | 0.255 | 1.101 | 0.933 | 1.301 |
|  | NPAR(2) | 0.172 | 0.086 | 4.002 | 1 | 0.045 | 1.188 | 1.003 | 1.407 |
|  | NPAR(3) | 0.251 | 0.087 | 8.310 | 1 | 0.004 | 1.285 | 1.084 | 1.524 |
|  | Constant | -5.419 | 0.242 | 502.312 | 1 | 0.000 | 0.004 |  |  |
| a. Variable(s) entered on step 1: gender, age, race/ethnicity, PIR, education, marital status, health insurance, tobacco use, alcohol use, hypertension, T2DM, cardiovascular disease, WC, PA, body mass index (BMI), TG, HDL, ALT, AST, GGT,and NPAR. | | | | | | | | | |

| **TABLE S5(A**) The Variables in the Equation for SII and MASLD after PSM | | | | | | | | | |
| --- | --- | --- | --- | --- | --- | --- | --- | --- | --- |
|  | | B | S.E. | Wald | df | Sig. | OR | 95% C.I.for OR | |
|  |  |  |  |  |  |  |  | Lower | Upper |
| Step 1^a^ | SII |  |  | 19.456 | 3 | 0.000 |  |  |  |
|  | SII (1) | 0.268 | 0.102 | 6.985 | 1 | 0.008 | 1.308 | 1.072 | 1.596 |
|  | SII (2) | 0.269 | 0.103 | 6.882 | 1 | 0.009 | 1.309 | 1.070 | 1.601 |
|  | SII (3) | 0.442 | 0.101 | 19.137 | 1 | 0.000 | 1.556 | 1.277 | 1.897 |
|  | Constant | -0.248 | 0.073 | 11.676 | 1 | 0.001 | 0.780 |  |  |
| a. Variable(s) entered on step 1: SII. | | | | | | | | | |

| **TABLE S5(B**) The Variables in the Equation for SIRI and MASLD after PSM | | | | | | | | | |
| --- | --- | --- | --- | --- | --- | --- | --- | --- | --- |
|  | | B | S.E. | Wald | df | Sig. | OR | 95% C.I.for OR | |
|  |  |  |  |  |  |  |  | Lower | Upper |
| Step 1^a^ | SIRI |  |  | 15.718 | 3 | 0.001 |  |  |  |
|  | SIRI(1) | 0.014 | 0.101 | 0.018 | 1 | 0.893 | 1.014 | 0.832 | 1.236 |
|  | SIRI(2) | 0.281 | 0.103 | 7.464 | 1 | 0.006 | 1.324 | 1.083 | 1.619 |
|  | SIRI (3) | 0.299 | 0.100 | 8.918 | 1 | 0.003 | 1.348 | 1.108 | 1.640 |
|  | Constant | -0.148 | 0.072 | 4.272 | 1 | 0.039 | 0.863 |  |  |
| a. Variable(s) entered on step 1: SIRI. | | | | | | | | | |

| **TABLE S5(C**) The Variables in the Equation for LMR and MASLD after PSM | | | | | | | | | |
| --- | --- | --- | --- | --- | --- | --- | --- | --- | --- |
|  | | B | S.E. | Wald | df | Sig. | OR | 95% C.I.for OR | |
|  |  |  |  |  |  |  |  | Lower | Upper |
| Step 1^a^ | LMR |  |  | 2.969 | 3 | 0.396 |  |  |  |
|  | LMR(1) | 0.146 | 0.101 | 2.110 | 1 | 0.146 | 1.158 | 0.950 | 1.410 |
|  | LMR(2) | 0.139 | 0.099 | 1.965 | 1 | 0.161 | 1.149 | 0.946 | 1.394 |
|  | LMR(3) | 0.139 | 0.103 | 1.818 | 1 | 0.178 | 1.149 | 0.939 | 1.405 |
|  | Constant | -0.105 | 0.071 | 2.208 | 1 | 0.137 | 0.900 |  |  |
| a. Variable(s) entered on step 1: LMR. | | | | | | | | | |

| **TABLE S5(D**) The Variables in the Equation for NLR and MASLD after PSM | | | | | | | | | |
| --- | --- | --- | --- | --- | --- | --- | --- | --- | --- |
|  | | B | S.E. | Wald | df | Sig. | OR | 95% C.I.for OR | |
|  |  |  |  |  |  |  |  | Lower | Upper |
| Step 1^a^ | NLR |  |  | 8.684 | 3 | 0.034 |  |  |  |
|  | NLR (1) | 0.207 | 0.100 | 4.229 | 1 | 0.040 | 1.230 | 1.010 | 1.497 |
|  | NLR (2) | 0.161 | 0.102 | 2.509 | 1 | 0.113 | 1.175 | 0.962 | 1.434 |
|  | NLR(3) | 0.287 | 0.101 | 8.160 | 1 | 0.004 | 1.333 | 1.094 | 1.623 |
|  | Constant | -0.164 | 0.071 | 5.303 | 1 | 0.021 | 0.849 |  |  |
| a. Variable(s) entered on step 1: NLR. | | | | | | | | | |

| **TABLE S5(E**) The Variables in the Equation for PLR and MASLD after PSM | | | | | | | | | |
| --- | --- | --- | --- | --- | --- | --- | --- | --- | --- |
|  | | B | S.E. | Wald | df | Sig. | OR | 95% C.I.for OR | |
|  |  |  |  |  |  |  |  | Lower | Upper |
| Step 1^a^ | PLR |  |  | 3.806 | 3 | 0.283 |  |  |  |
|  | PLR(1) | 0.182 | 0.102 | 3.182 | 1 | 0.074 | 1.199 | 0.982 | 1.464 |
|  | PLR(2) | 0.159 | 0.101 | 2.499 | 1 | 0.114 | 1.173 | 0.963 | 1.429 |
|  | PLR(3) | 0.125 | 0.102 | 1.513 | 1 | 0.219 | 1.133 | 0.928 | 1.383 |
|  | Constant | -0.117 | 0.072 | 2.637 | 1 | 0.104 | 0.889 |  |  |
| a. Variable(s) entered on step 1: PLR. | | | | | | | | | |

| **TABLE S5(F**) The Variables in the Equation for NPAR and MASLD after PSM | | | | | | | | | |
| --- | --- | --- | --- | --- | --- | --- | --- | --- | --- |
|  | | B | S.E. | Wald | df | Sig. | OR | 95% C.I.for OR | |
|  |  |  |  |  |  |  |  | Lower | Upper |
| Step 1^a^ | NPAR |  |  | 6.846 | 3 | 0.077 |  |  |  |
|  | NPAR (1) | 0.076 | 0.102 | 0.550 | 1 | 0.458 | 1.079 | 0.883 | 1.317 |
|  | NPAR(2) | 0.205 | 0.103 | 3.968 | 1 | 0.046 | 1.228 | 1.003 | 1.503 |
|  | NPAR(3) | 0.230 | 0.102 | 5.127 | 1 | 0.024 | 1.259 | 1.031 | 1.536 |
|  | Constant | -0.130 | 0.074 | 3.118 | 1 | 0.077 | 0.878 |  |  |
| a. Variable(s) entered on step 1: NPAR. | | | | | | | | | |

| **Table S6** Association between systemic immune biomarkers and Advanced fibrosis | | | | |
| --- | --- | --- | --- | --- |
| Variables | OR | 95% CI | | P-value |
|  |  | Lower | Upper |  |
| **SII** |  |  |  |  |
| quartile 1 | Reference |  |  |  |
| quartile 2 | 0.423 | 0.330 | 0.543 | 0.000 |
| quartile 3 | 0.376 | 0.292 | 0.483 | 0.000 |
| quartile 4 | 0.218 | 0.167 | 0.284 | 0.000 |
| **SIRI** |  |  |  |  |
| quartile 1 | Reference |  |  |  |
| quartile 2 | 0.864 | 0.662 | 1.127 | 0.281 |
| quartile 3 | 0.758 | 0.581 | 0.987 | 0.040 |
| quartile 4 | 1.030 | 0.796 | 1.333 | 0.822 |
| **LMR** |  |  |  |  |
| quartile 1 | Reference |  |  |  |
| quartile 2 | 0.616 | 0.484 | 0.784 | 0.000 |
| quartile 3 | 0.674 | 0.529 | 0.858 | 0.001 |
| quartile 4 | 0.639 | 0.488 | 0.838 | 0.001 |
| **NLR** |  |  |  |  |
| quartile 1 | Reference |  |  |  |
| quartile 2 | 0.665 | 0.506 | 0.873 | 0.003 |
| quartile 3 | 0.966 | 0.746 | 1.251 | 0.793 |
| quartile 4 | 1.091 | 0.850 | 1.402 | 0.493 |
| **PLR** |  |  |  |  |
| quartile 1 | Reference |  |  |  |
| quartile 2 | 1.752 | 1.329 | 2.310 | 0.000 |
| quartile 3 | 2.259 | 1.717 | 2.971 | 0.000 |
| quartile 4 | 5.219 | 4.008 | 6.797 | 0.000 |
| **NPAR** |  |  |  |  |
| quartile 1 | Reference |  |  |  |
| quartile 2 | 1.144 | 0.862 | 1.517 | 0.351 |
| quartile 3 | 1.374 | 1.050 | 1.798 | 0.021 |
| quartile 4 | 2.407 | 1.856 | 3.121 | 0.000 |
